# IL-1 receptor antagonist ameliorates inflammasome-dependent inflammation in murine and human cystic fibrosis

**DOI:** 10.1038/ncomms10791

**Published:** 2016-03-14

**Authors:** Rossana G. Iannitti, Valerio Napolioni, Vasilis Oikonomou, Antonella De Luca, Claudia Galosi, Marilena Pariano, Cristina Massi-Benedetti, Monica Borghi, Matteo Puccetti, Vincenzina Lucidi, Carla Colombo, Ersilia Fiscarelli, Cornelia Lass-Flörl, Fabio Majo, Lisa Cariani, Maria Russo, Luigi Porcaro, Gabriella Ricciotti, Helmut Ellemunter, Luigi Ratclif, Fernando Maria De Benedictis, Vincenzo Nicola Talesa, Charles A. Dinarello, Frank L. van de Veerdonk, Luigina Romani

**Affiliations:** 1 Department of Experimental Medicine, University of Perugia, 06132 Perugia, Italy; 2 Unit of Endocrinology and Diabetes, Bambino Gesù Children's Hospital, 00165 Rome, Italy; 3 Fondazione IRCCS Ca' Granda, Ospedale Maggiore Policlinico, University of Milan, 20122 Milan, Italy; 4 Children's Hospital Bambino Gesù IRCCS, 00165 Rome, Italy; 5 Division of Hygiene and Medical Microbiology, Innsbruck Medical University, 6020 Innsbruck, Austria; 6 CF Centre, Medical University Innsbruck, 6020 Innsbruck, Austria; 7 Servizio di Supporto Fibrosi Cistica, Istituto Ospedale G. Tatarella, Foggia, 71042 Cerignola, Italy; 8 Department of Mother and Child Health, Salesi Children's Hospital, 60128 Ancona, Italy; 9 Radboud Center for Infectious Diseases, Nijmegen, 6500 HB, The Netherlands; 10 Division of Infectious Diseases, University of Colorado Denver, Aurora, Colorado 80045, USA; 11 Department of Internal Medicine, Radboud Center for Infectious diseases (RCI), Radboudumc, Nijmegen, 6500 HB, The Netherlands

## Abstract

Dysregulated inflammasome activation contributes to respiratory infections and pathologic airway inflammation. Through basic and translational approaches involving murine models and human genetic epidemiology, we show here the importance of the different inflammasomes in regulating inflammatory responses in mice and humans with cystic fibrosis (CF), a life-threatening disorder of the lungs and digestive system. While both contributing to pathogen clearance, NLRP3 more than NLRC4 contributes to deleterious inflammatory responses in CF and correlates with defective NLRC4-dependent IL-1Ra production. Disease susceptibility in mice and microbial colonization in humans occurrs in conditions of genetic deficiency of NLRC4 or IL-1Ra and can be rescued by administration of the recombinant IL-1Ra, anakinra. These results indicate that pathogenic NLRP3 activity in CF could be negatively regulated by IL-1Ra and provide a proof-of-concept evidence that inflammasomes are potential targets to limit the pathological consequences of microbial colonization in CF.

## Abstract (web summary)

IL-1-mediated inflammation contributes to the pathogenesis of cystic fibrosis. Here the authors show that this is largely due to NLRP3 activation, whereas NLRP4 induces IL-1Ra, limiting the overall inflammasome activity and providing a therapeutic angle to ameliorate the disease.

## Graphical abstract

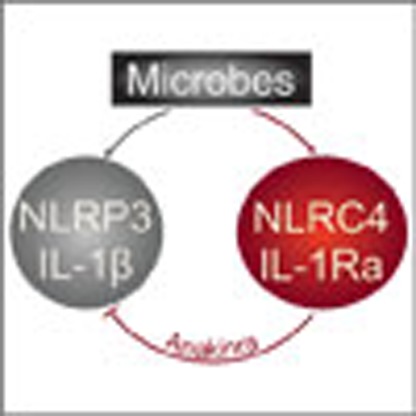

In patients with cystic fibrosis (CF), a vicious cycle of airways infection, inflammation and tissue damage is responsible for the progressive decline of pulmonary function.The pulmonary innate response in CF is dysregulated at several levels, resulting in inefficient bacterial clearance and contributing to lung disease associated with CF^1^. Studies have documented an altered balance of inflammatory/anti-inflammatory cytokines in CF^2^, providing evidence that targeting specific inflammatory/anti-inflammatory pathways is a valid therapeutic strategy in CF^3^,^4^.

Dysregulated inflammasome activity is a key mediator of infections and airway inflammation in lung diseases^5^. Indeed, NLRP3 inflammasome activity is involved in host response to acute lung infections, but also during progression of several chronic pulmonary diseases^6^. Assembly of these intracellular danger sensors triggers pyroptosis and secretion of bioactive interleukin (IL)-1β and IL-18 that are central to processes mediating lung inflammation^5^. Both bronchial epithelial cells (ECs)^7^ and haematopoietic cells^8^ are source of IL-1β production in CF (between 2.8 and 32 ng ml^−1^ in sputum from CF children)^9^. IL-1 signalling triggers the activation of pathogenic IL-17A-secreting T cells, thus critically modulating the Th17/ regulatory T (Treg) cell balance^10^. This balance is essential for the efficient control of *Aspergillus fumigatus* colonization and diseases in CF^3^, where the colonization by the fungus is common and may lead to fungal sensitization, bronchitis, allergic bronchopulmonary aspergillosis (ABPA)^11^ and FEV1 worsening^12^. Pathogenic Th17 cells accounted for the inherent susceptibility to aspergillosis in CF due to an exuberant inflammatory response that compromises the host's ability to control the infection^3^. Preventing NLRP3 activation and reducing IL-1β secretion reduced infection severity in chronic granulomatous disease^13^ as well as CF^14^. Thus, despite the key role in host protection against the fungus^14^,^15^, the inflammasome/IL-1 pathway is tightly regulated to avoid an excessive inflammatory pathology^16^.

Similarly, despite a clear protective role for IL-1R signalling^7^ and NLRC4 (refs 17, 18, 19) in the innate immunity against *Pseudomonas aeruginosa*, the most common pathogen in CF, deregulated inflammasome signalling also aggravates *P. aeruginosa* pneumonia in CF^20^ and non-CF^21^ conditions. The fact that *P. aeruginosa* has adopted mechanisms to inhibit NLRC4-mediated caspase-1 activation^22^ further suggests the importance of this pathway against Gram-negative bacterial pneumonia. Thus, the inflammasome may represent a potential target to limit the pathological consequences of pulmonary infections in CF.

In the present study, we determine the relative contribution of different inflammasomes to infection and inflammation in CF mice, assessed the therapeutic efficacy of the recombinant IL-1R antagonist (IL-1Ra), anakinra, and evaluated whether genetic variations in the inflammasome/IL-1RI signalling could contribute to microbial colonization and inflammation in human CF. We found that NLRP3 more than NLRC4 contributes to IL-1β-dependent inflammation in murine and human CF. Pathogenic NLRP3 activity, however, is negatively regulated by IL-1Ra, thus providing a therapeutic angle to ameliorate the pathological consequences of microbial colonization in CF.

## Results

### Dysregulated inflammasome activity in murine CF

**Figure 1:**
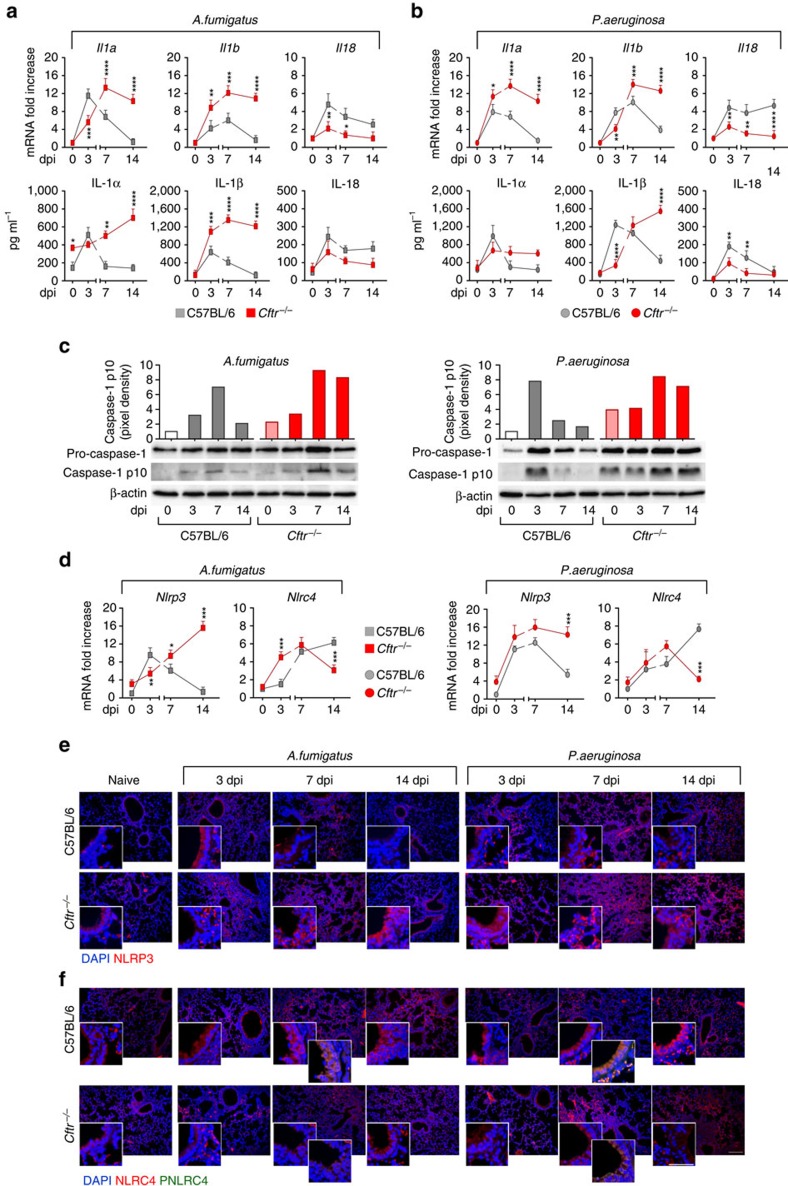
Dysregulated inflammasome activity in murine CF. C57BL/6 and *Cftr*^*−/−*^mice (*n*=6 for all groups) were infected intranasally with live *A. fumigatus* conidia or *P. aeruginosa* and assessed for IL-1β, IL-18 and IL-1α gene expression and cytokine production in lung homogenates of *A. fumigatus-* (**a**) or *P. aeruginosa-* (**b**) infected mice at different days post-infection (dpi) by RT–PCR and specific ELISA; (**c**) Caspase-1 cleavage by immunoblotting with specific antibodies and corresponding pixel density ratio normalized against corresponding β-actin; *Nlrp3* and *Nlrc4* gene expression (**d**) by RT–PCR in lung tissues and protein expression (**e**,**f**) by lung immunofluorescence staining with anti-NLRP3 antibody followed by anti-rabbit TRICT (**e**) and (**f**) anti-NLRC4 antibody and anti-phospho(p)NLRC4 followed by anti-rabbit TRICT and anti-hamster FITC. Cell nuclei were stained blue with DAPI. Representative images were acquired with a high-resolution Microscopy Olympus DP71 with a × 20 objective. Scale bars, 200 μm, inset 50 μm. Note the expression on lung epithelium cells in the inset. Scanning densitometry was done with Image Lab 3.1.1 software. Data are representative (immunoblotting) or pooled from three experiments and presented as mean±s.d. for all bar graphs. *P<0.05, **P<0.01, ***P<0.001, ****P<0.0001, C57BL/6 versus *Cftr*^*−/−*^mice at different dpi, Two-way ANOVA, Bonferroni *post hoc* test. For NLRP3 or NLRC4 quantification, see Supplementary Fig. 1.

**Figure 2:**
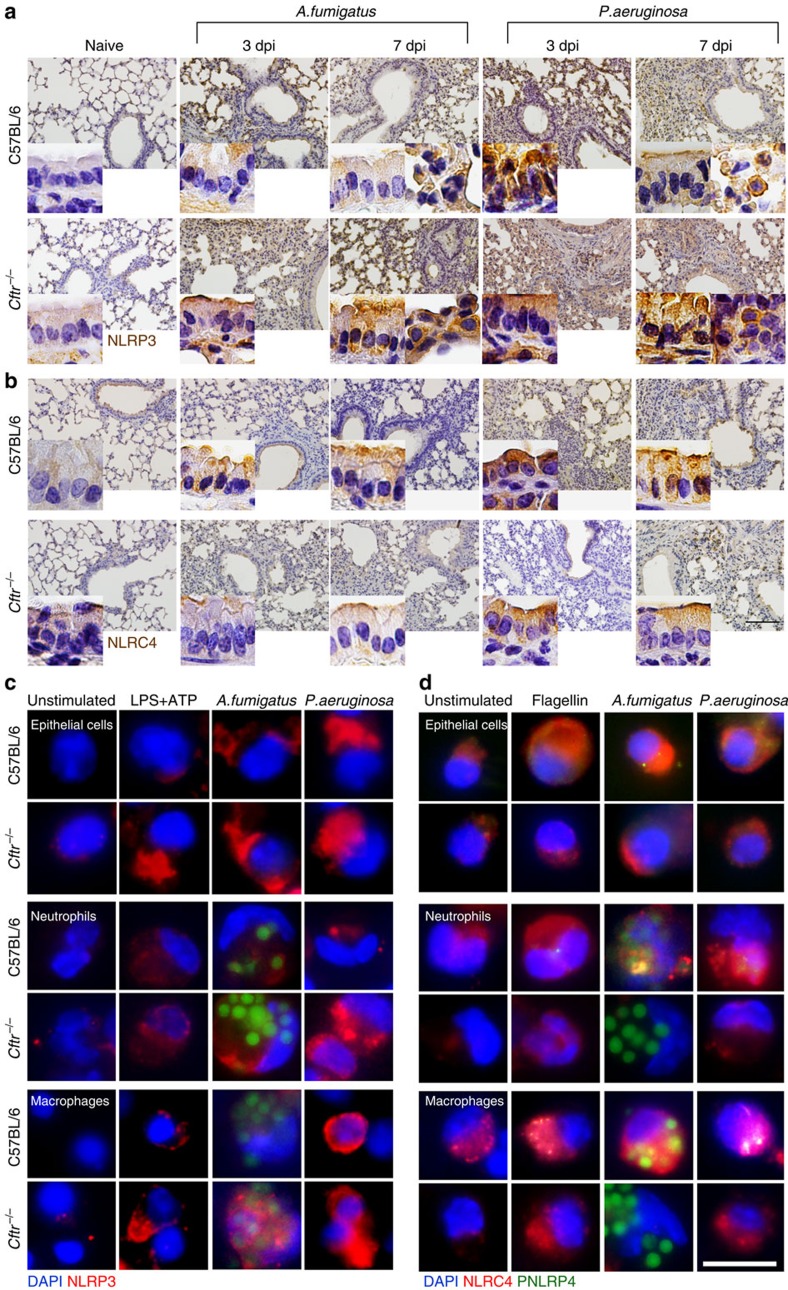
Different NLRP3 and NLRC4 expression in lung cells from CF mice. C57BL/6 and *Cftr*^*−/−*^mice (*n*=6 for all groups) were infected intranasally with live *A. fumigatus* conidia or *P. aeruginosa* and assessed for (**a**) NLRP3 and (**b**) NLRC4 protein expression in the lungs and lung epithelial and myeloid cells (magnified in the insets) by immunohistochemistry. Cell nuclei were counterstained with haematoxylin. Representative images of two independent experiments were acquired with a × 20 and × 60 (inset, using EVOS FL Color Imaging System) objective. Scale bar, 200 μm. Immunofluorescence staining with (**c**) NLRP3 followed by anti-rabbit TRICT or (**d**) pNLRC4 and NLRC4 of epithelia cells and macrophages purified from lungs and neutrophils from the peritoneal cavity of C57BL/6 and *Cftr*^*−/−*^ uninfected mice exposed *in vitro* to LPS+ATP, flagellin, *A. fumigatus* live conidia or *P. aeruginosa.* Cell nuclei were counterstained blue with DAPI. Images were acquired a high-resolution Microscopy Olympus DP71 using a × 100 objective. Scale bar, 12.5 μm. For number of cells with positive NLRP3 or NLRC4 expression quantification, see Supplementary Fig. 1.

To evaluate inflammasome activity in murine CF, we infected *Cftr*^*–/–*^ and C57BL/6 mice intranasally with *A. fumigatus* or intratracheally with *P. aeruginosa* and evaluated IL-1β, IL-1α and IL-18 production, caspase-1 cleavage and expression of NLRP3 or NLRC4 for their involvement in response to both *A. fumigatus*^23^ and *P. aeruginosa*^17^,^18^,^19^,^20^,^21^,^22^. We found a significant increased production of IL-1α and IL-1β, but not IL-18, (Fig. 1a,b) and of the cleaved fragment of procaspase-1 (Fig. 1c) in the lungs of *Cftr*^*–/–*^ than C57BL/6 mice during either infection. On assessing NLRP3 or NLRC4 gene (Fig. 1d) or protein (Fig. 1e) expression, it was found that NLRP3 expression was robust during the first week of either infection with a decline thereafter. In contrast, the expression of NLRC4 persistently increased in C57BL/6 mice through the infection. Differently from what observed in C57BL/6 mice, NLRP3 expression persistently increased in *Cftr*^*–/–*^ mice, while NLRC4 expression was not sustained (Fig. 1d,e and Supplementary Fig. 1). Confirming previous findings^24^, AIM2 was upregulated in infection and particularly in *Cftr*^*–/–*^ mice, while NLRP6 was not (Supplementary Fig. 2). As phosphorylation on Ser 533 by PKCδ could be required for NLRC4 activity^25^, we did immunostaining with a specific anti-phospho (p)NLRC4 antibody to reveal that pNLRC4 expression paralleled that of NLRC4 on ECs from C57BL/6 mice but not *Cftr*^*–/–*^ mice in which pNLRC4 could not be detected (Fig. 1e). Immunohistochemistry of the lungs (Fig. 2a,b) and studies done on purified cells isolated from naive lungs (Fig. 2c,d) confirmed that NLRP3 expression was higher in ECs and myeloid (alveolar macrophages and neutrophils) cells from *Cftr*^*–/–*^than C57BL/6 mice, while pNLRC4 expression was inherently lower, particularly in *Cftr*^*–/–*^ECs (quantified in Supplementary Fig. 1). These results indicate that NLRP3 and NLRC4 are differentially regulated during infection in *Cftr*^*–/–*^mice compared with C57BL/6 mice, pNLRC4 activation being defective in *Cftr*^*–/–*^ECs.

**Figure 3:**
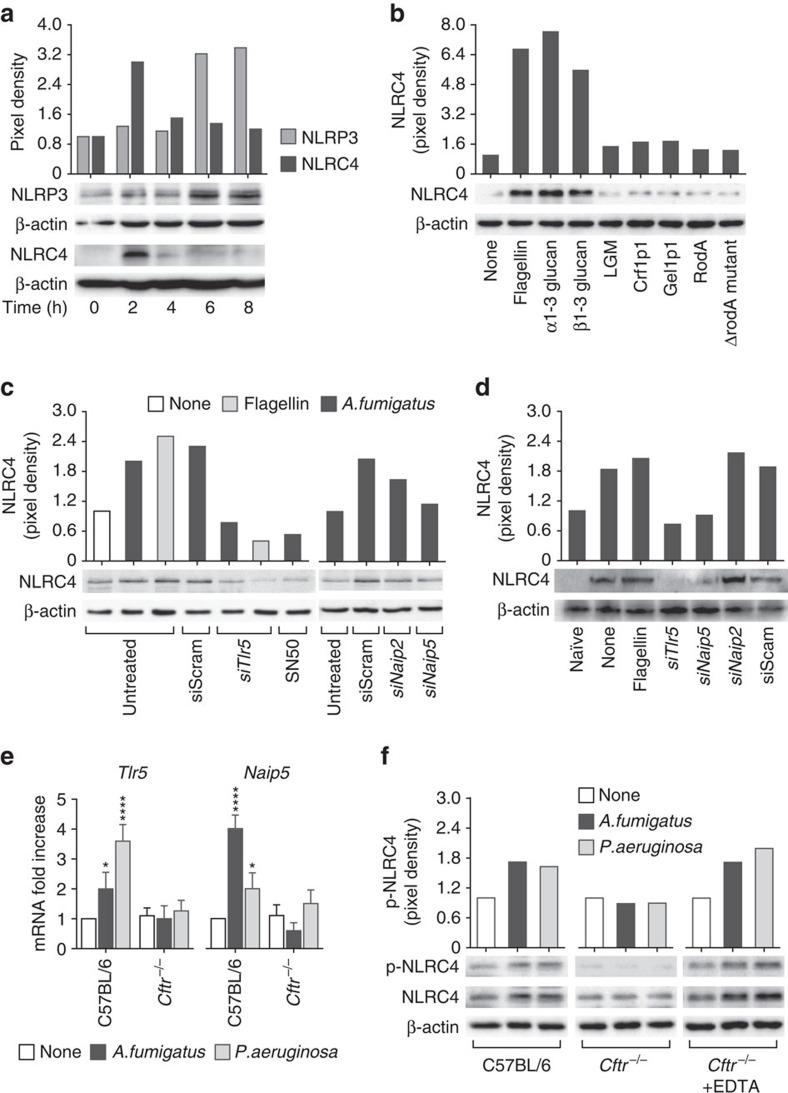
The TLR5/NAIP5 pathway of NLRC4 activation is defective in murine CF. RAW 247.3 macrophages were exposed to (**a**) *A. fumigatus* live conidia before the assessment for NLRP3 or NLRC4 protein expression at different times by immunoblotting with specific antibodies or to (**b**) various fungal antigens for 2 h at 37 °C before NLRC4 protein expression by western blotting. (**c**) Cells were pretreated with specific siRNA for *Tlr5*, *Naip2*, *Naip5* or scrambled siRNA (siScram) or the NF-κB inhibitor SN50 (100 μM), exposed to *Aspergillus* conidia or flagellin for 2 h at 37 °C and assessed for NLRC4 protein expression by western blotting. All immunoblots were normalized against the corresponding β-actin. (**d**) C57BL/6 mice were given siRNA intranasally twice, 2 days before and 3 days after the infection before the assessment of NLRC4 protein expression at 4 dpi. (**e**) *Tlr5*, *Naip2* and *Naip5* expression by RT–PCR in lungs of C57BL/6 and *Cftr*^*−/−*^ mice infected with *A. fumigatus* or *P. aeruginosa* (*n*=6 for all groups) 3 days before. (**f**) NLRC4 and phospho(p)NLRC4 expression in purified lung marcrophages from C57BL/6 and *Cftr*^*−/−*^ mice stimulated with live *A. fumigatus* conida or *P. aeruginosa* at the ratio cells:microbes (1:1) in the presence of EDTA at 37 °C. The expression of pNLRC4 was normalized against the corresponding β-actin. Data pooled from three experiments and presented as mean±s.d. for all bar graphs. *P<0.05, ***P<0.001, ****P<0.0001, None versus infected mice, Two-way ANOVA, Bonferroni *post hoc* test.

At variance with NLRP3, whose activation by *A. fumigatus* hyphal fragments has been reported^23^, the activation of NLRC4 by the fungus is a novel finding that deserved further investigation. On assessing the kinetics of protein expression of NLRC4 and NLRP3 in RAW245.7 cells exposed to live conidia and/or several fungal molecules, we found that NLRC4 was maximally expressed after 2 h of exposure to decline thereafter (Fig. 3a) at the time at which NLRP3 expression was maximally induced (Fig. 3a); fungal polysaccharides (both α-and β-glucan) and not other cell surface proteins or lipoproteins activated NLRC4 (Fig. 3b), a finding suggesting that NLRC4 activation likely occurs in concomitance with conidia germination, while NLRP3 is activated by escaping hyphae. Activation of NLRC4 can be mediated by cytosolic bacterial flagellin^26^,^27^ and/or its cognate recognition by TLR5 (ref. 19) and involves members of the immune sensors NAIPs (NLR family, apoptosis inhibitory proteins) family^28^. By resorting to siRNA-mediated gene knockdown *in vitro* (Fig. 3c) and *in vivo* (Fig. 3d), we found that the activation of NLRC4 in response to the fungus was, similar to flagellin, both TLR5- and NAIP5-dependent and involved NF-κB signalling and not NAIP2. Both *Tlr5* and *Naip5* expressions failed to upregulate in the lungs of *Cftr*^*–/–*^ as opposed to C57BL/6 mice (Fig. 3e), a finding that may account for the defective NLRC4 activation in CF. However, the defective NLRC4 phosphorylation in CF could be restored by blocking the increased calcium signalling of CF airway epithelia (Fig. 3f)^29^, a finding pointing to the complexity of NLRC4 activation *in vivo* during infection. Indeed, despite similarity, the activation of NLRC4 in response to *A. fumigatus* or *P. aeruginosa* appears to be different, as A/J mice were as susceptible as NLRC4-deficient mice to *A. fumigatus* but not to *P. aeruginosa* infection (Supplementary Fig. 3a).

### NLRP3 and NLRC4 activity is non-redundant in lung infections

**Figure 4:**
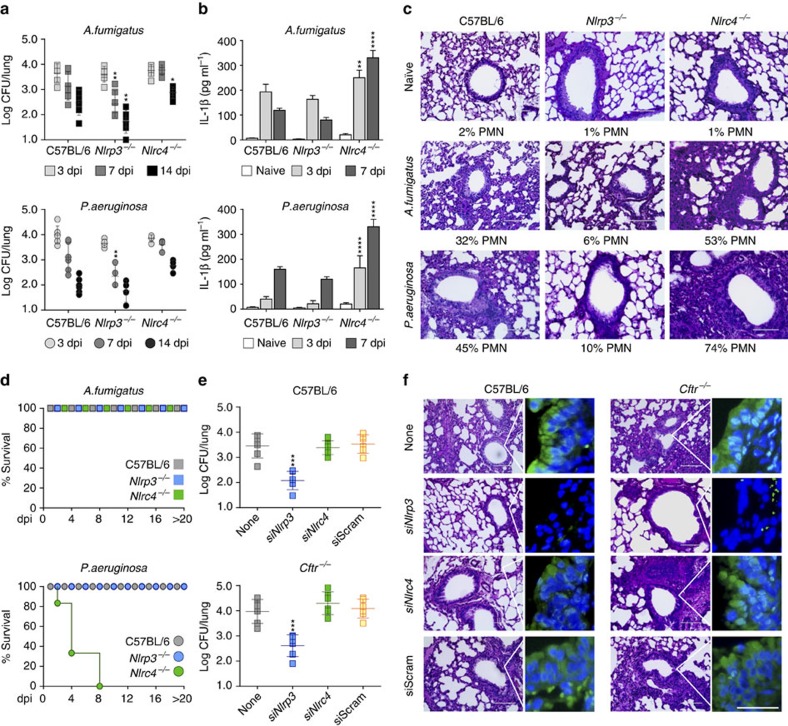
NLRP3 and NLRC4 are non-redundantly activated in lung infections. C57BL/6, *Nlrp3*^*−/−*^ and *Nlrc4*^*−/−*^ mice (*n*=6 for all groups) were infected intranasally with live *A. fumigatus* conidia or *P. aeruginosa* and assessed for (**a**) fungal or bacterial growth at different dpi; (**b**) IL-1β production in BAL fluids (**c**) and lung histology at 7 dpi (periodic acid-Schiff staining) (% of neutrophils in the bronchoalveolar lavage are shown in the insets). (**d**) Survival, (**e**) fungal growth and (**f**) lung histology of C57BL/6 and *Cftr*^*−/−*^mice infected with *A. fumigatus* conidia and treated with specific *Nlrp3*, *Nlrc4* siRNA or scrambled siRNA. Fungal growth (log CFU, mean±s.d.) and histology were assessed at 7 dpi In **f**, periodic acid-Schiff staining and increased deposition of DNA on lung parenchyma cells on TUNEL staining. Cell nuclei were stained blue with DAPI. Representative images of two independent experiments were acquired using EVOS FL Color Imaging System with a × 40 objective for histology (Scale bar, 100 μm) and a high-resolution Microscopy Olympus DP71 using a × 20 objective for TUNEL (Scale bar, 50 μm). Data pooled from three experiments and presented as mean±s.d. for all bar graphs. *P<0.05, **P<0.01, ***P<0.001, ****P<0.0001, C57BL/6 versus *Nlrp3*^*−/−*^*, Nlrc4*^*−/−*^or *Cftr*^*−/−*^mice at different dpi (**a**,**b**) or untreated (none) versus siRNA treated mice (**e**), Two-way (**a**,**b**) and One-way ANOVA (**e**) Bonferroni *post hoc* test.

To unravel the functional activity of either NLRP3 or NLRC4 in these infection models, we assessed *Nlrp3*^–/–^ or *Nlrc4*^*–/–*^ mice for susceptibility to either infection and inflammasome activity. *Nlrp3*^*–/–*^ mice showed increased resistance to either infection, as revealed by the reduced fungal or bacterial load (Fig. 4a), IL-1β production (Fig. 4b) and lung inflammatory cell recruitment (Fig. 2c). Only when infected with a high number of *Aspergillus* conidia were *Nlrp3*^*–/–*^ mice more susceptible and unable to restrict fungal growth and inflammatory pathology (Supplementary Fig. 4). In contrast, *Nlrc4*^*–/–*^ mice were less efficient in restraining either the fungal or bacterial growth (Fig. 4a), despite elevated IL-1β production (Fig. 4b). Moreover, these mice were unable to control the local inflammatory cell recruitment (Fig. 4c) and succumbed to the *P. aeruginosa* infection (Fig. 4d). In addition, blocking *Nlrp3* with siRNA increased resistance to *Aspergillus* infection in both C57BL/6 and *Cftr*^*–/–*^ mice, as revealed by decreased lung colony-forming units (CFUs; Fig. 4e) and pathology as well as decreased positive staining for terminal deoxynucleotidyl transferase-mediated deoxyuridine triphosphate nick-end labelling (TUNEL; Fig. 4f), a marker of both apoptotic and pyropoptotic cell death^30^. In contrast, blocking *Nlrc4* did not affect the susceptibility of C57BL/6 or *Cftr*^*–/–*^ mice (Fig. 4e,f). Similar results were obtained with *P. aeruginosa* infection (Supplementary Fig. 5). These data suggest that NLRP3 and NLRC4, despite having overlapping functions, may have a different role in these lung infections whereby NLRP3 may contribute to pathogenic inflammation in the relative absence of NLRC4. As such, these results are consistent with the high or low susceptibility to bacterial or fungal pneumonia observed in mice with a high or low inflammasome activity, such as *Il1r8*^*–/–*^ and *Il1r1*^*–/–*^ mice, respectively^14^,^21^,^31^,^32^ in which we found that NLRP3 expression was more robust in the former than in the latter (Supplementary Fig. 6). Thus, IL-1RI signalling leading to NLRP3 activation may promote deleterious inflammation in response to *A. fumigatus* and *P. aeruginosa* in CF.

### NLRC4 induces IL-1Ra that dampens NLRP3 activity

**Figure 5:**
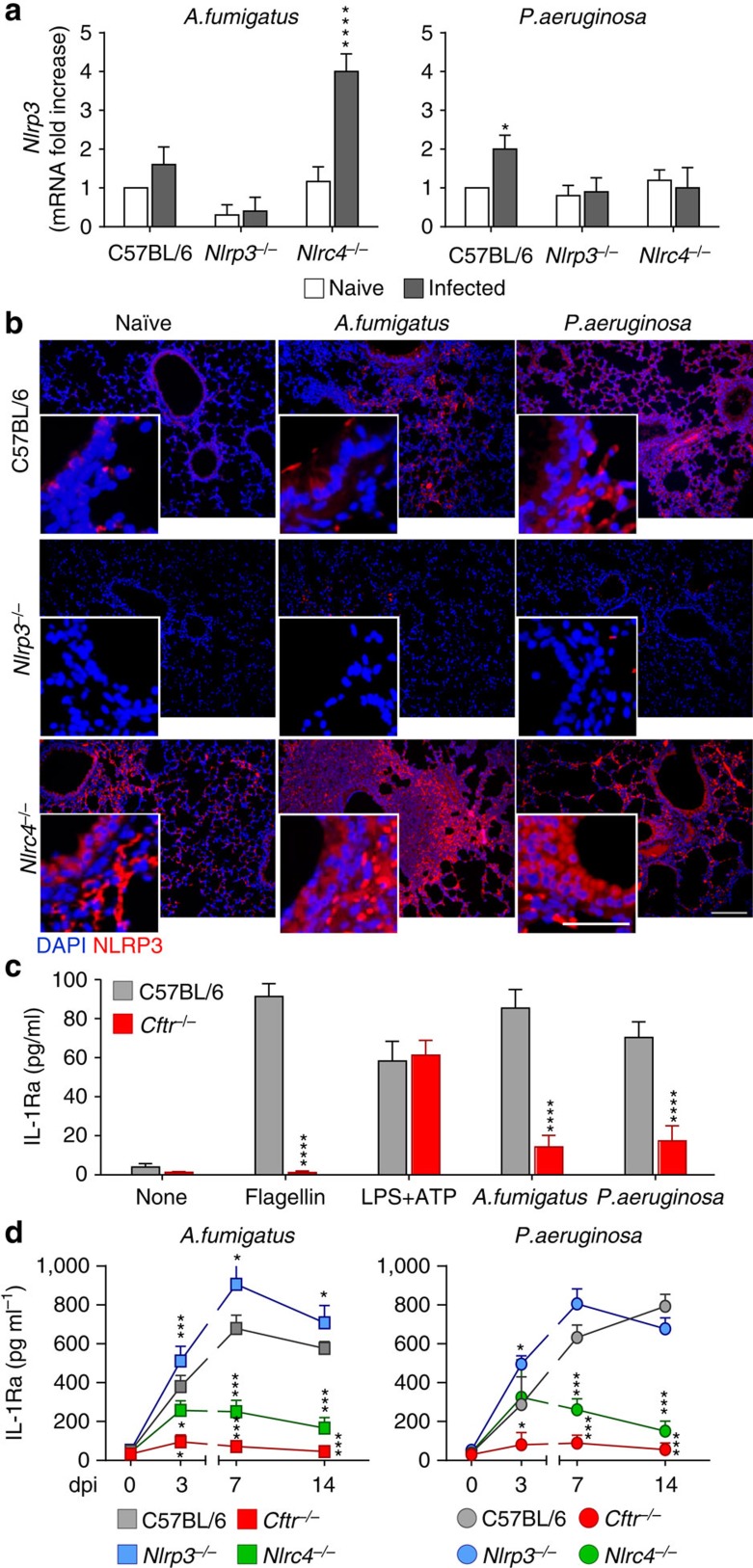
NLRC4 produces IL-1Ra. C57BL/6, *Nlrp3*^*−/−*^*, Nlrc4*^*−/−*^ and *Cftr*^*−/−*^ mice (*n*=6 for all groups) were infected intranasally with live *A. fumigatus* conidia or *P. aeruginosa.* NLRP3 gene (**a**) and protein (**b**) expression by RT–PCR or immunofluorescence staining (anti-NLRP3 antibody followed by anti-goat TRICT secondary antibody) of lungs at 7 dpi. Cell nuclei were stained blue with DAPI. Representative images of two independent experiments were acquired with a high-resolution Microscopy Olympus DP71 using a × 20 objective. Scale bar, 200 μm, inset 50 μm. IL-1Ra production (quantified by specific ELISA) in (**c**) supernatants of purified lung epithelial cells exposed to the different stimuli, with and without 2 mM of EDTA for 6 h at 37 °C and (**d**) lung homogenates at different dpi. Data are representative (immunofluorescence) or pooled from three experiments and presented as mean±s.d. for all bar graphs. *P<0.05, **P<0.01, ***P<0.001, ****P<0.0001, C57BL/6 versus *Nlrp3*^*−/−*^*, Nlrc4*^*−/−*^ and *Cftr*^*−/−*^ mice at different dpi. Two-way ANOVA Bonferroni *post hoc* test. For NLRP3 quantification, see Supplementary Fig. 1.

**Figure 6:**
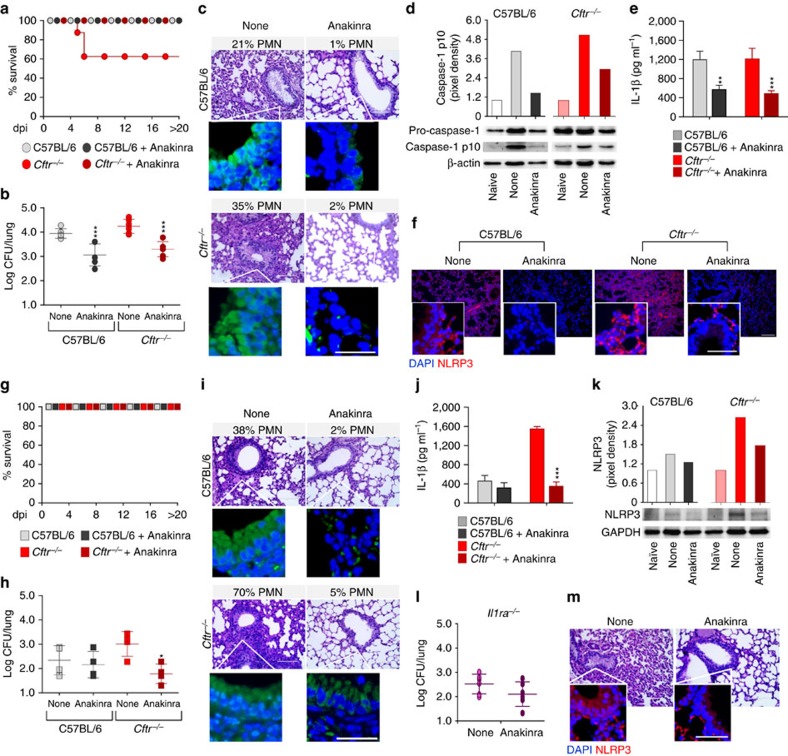
Anakinra protects *Cftr*^*−/−*^mice from infections and NLRP3 inflammation. C57BL/6 and *Cftr*^−/−^ mice (*n*=6 for all groups) were infected intranasally with live *A. fumigatus* conidia or *P. aeruginosa* and treated with anakinra (100 mg kg^−1^ per day) throughout the infection. (**a**,**g**) Survival, (**b**,**h**) microbial growth (log CFU, mean±s.d.), (**c**,**i**) lung histology (periodic acid-Schiff staining) and reduced deposition of DNA on lung parenchyma cells by TUNEL; (**d**) caspase-1 cleavage by immunoblotting with specific antibodies (scanning densitometry was done with Image Lab 3.1.1 software. Representative of three independent experiments and corresponding pixel density ratio normalized against actin); (**e**,**j**) IL-1β levels in lung homogenates; NLRP3 protein expression by immunofluorescence staining (**f**) and immunoblotting (**k**) of lungs of anakinra-treated mice. Assays were done at 7 dpi. (**l**) Fungal growth (log CFU, mean±s.d.) and (**m**) lung histology (periodic acid-Schiff staining and NLRP3 immunofluorescence staining in the inset) of *A. fumigatus*-infected and anakinra-treated *Il1ra*^−/−^ mice. Representative images of two independent experiments were acquired using EVOS FL Color Imaging System with a × 40 objective for histology (Scale bar, 100 μm) and a high-resolution Microscopy Olympus DP71 using a × 20 objective for TUNEL and immunofluorescent staining (Scale bar, 200 μm, inset 50 μm). Data pooled from three experiments and presented as mean±s.d. for all bar graphs. *P<0.05, **P<0.01, ***P<0.001, anakinra treated versus untreated mice (none), One-way ANOVA (**b**,**h**), Two-way ANOVA (**e**,**j**) Bonferroni *post hoc* test and two-sides Student's *t*-test. (**l**) For NLRP3 quantification, see Supplementary Fig. 1.

The protective action of NLRC4 in Gram-negative bacterial pneumonia is mostly carried out in cooperation with NLRP3 to drive robust IL-1β signalling^21^,^33^. However, the precise mechanism(s) by which NLRC4 mediates its effects in the lung remains to be determined. IL-1Ra is a potent suppressor of inflammasome activity^34^. Although most intracellular IL-1Ra remains in the cytoplasm of cells, some isoforms may be released from airways ECs in some conditions and may act as extracellular receptor antagonists of IL-1RI (ref. 35). We have evidence that NLRC4 activation in response to *Candida albicans* resulted in a sustained production of IL-1Ra capable of restraining NLRP3 activity^36^. Should this mechanism be operative in the lung, this would suggest that NLRP3 expression is sustained in *Nlrc4*^*–/–*^ as well as *Il1ra*^*–/–*^ mice and that NLRC4 contributes to IL-1Ra production in either bacterial or fungal pneumonia. We observed a robust *Nlrp3* gene (Fig. 5a) and protein (Fig. 5b) expression in *Nlrc4*^*–/–*^ or *Il1ra*^*–/–*^ (Fig. 6m) infected mice as well as the major contribution of NLRC4 to IL-1Ra production. Indeed, the levels of IL-1Ra were sustained *in vitro* (Fig. 5c) and through the course of either infection *in vivo* (Fig. 5d) in C57BL/6 but not *Cftr*^*–/–*^ mice and NLRC4, more than NLRP3, contributed to this sustained production (Fig. 5d). Accordingly, IL-1Ra was deficient upon blocking TLR5 and NAIP5, both *in vitro* and *in vivo* (Supplementary Fig. 7a,b), as well as in A/J mice (Supplementary Fig. 3b) and was associated with increased IL-1β production and inflammatory responses. The opposing findings observed with flagellin (Supplementary Fig. 7a,b), further suggest the protective role of the TLR5/NLRC4 axis in infection. Altogether, these results suggest that NLRC4 and IL-1Ra deficiencies may contribute to NLRP3-mediated pathogenic inflammation in CF and that limiting NLRP3 via IL-1Ra could be of benefit in CF mice.

### Anakinra protects from infections and inflammation

These observations prompted us to investigate whether anakinra would ameliorate lung inflammatory pathology in CF. We treated C57BL/6 and *Cftr*^*–/–*^ mice infected with either *Aspergillus* or *Pseudomonas* with 10 mg kg^−1^ anakinra, a dose known to mimic human therapeutic dosages and to be pharmacologically active in mice^13^,^34^. Anakinra significantly increased survival of *Cftr*^*–/–*^ mice to *P. aeruginosa* infection (Fig. 6a) while reducing bacterial burden (Fig. 6b), neutrophil recruitment and lung damage (Fig. 6c). In addition, anakinra reduced caspase-1 cleavage (Fig. 6d), IL-1β production (Fig. 6e) and lung NLRP3 expression (Fig. 6f). Similar results were obtained in *Aspergillus*-infected *Cftr*^*–/–*^mice (Fig. 6g–k). Of interest, anakinra greatly increased the resistance to *A. fumigatus* infection (Fig. 6l), while decreasing NLRP3 expression (Fig. 6m), of the highly susceptible *Il1ra*^*–/–*^ mice. Mechanistically, regulation of NLRP3 expression by anakinra occurs at posttranslational level via the ubiquitin proteasome system (Supplementary Fig. 8), a degradation pathway known to regulate NLRP3 half life^37^. Altogether, these results indicate that by impairing neutrophil recruitment, anakinra may ameliorate lung pathology, without adversely affecting pathogen clearance. Of great interest, anakinra treatment was apparently more potent than the inhibition of IL-1β by a neutralizing antibody (Supplementary Fig. 9), a finding suggesting that the beneficial effect of anakinra may go beyond inflammasome inhibition.

**Figure 7:**
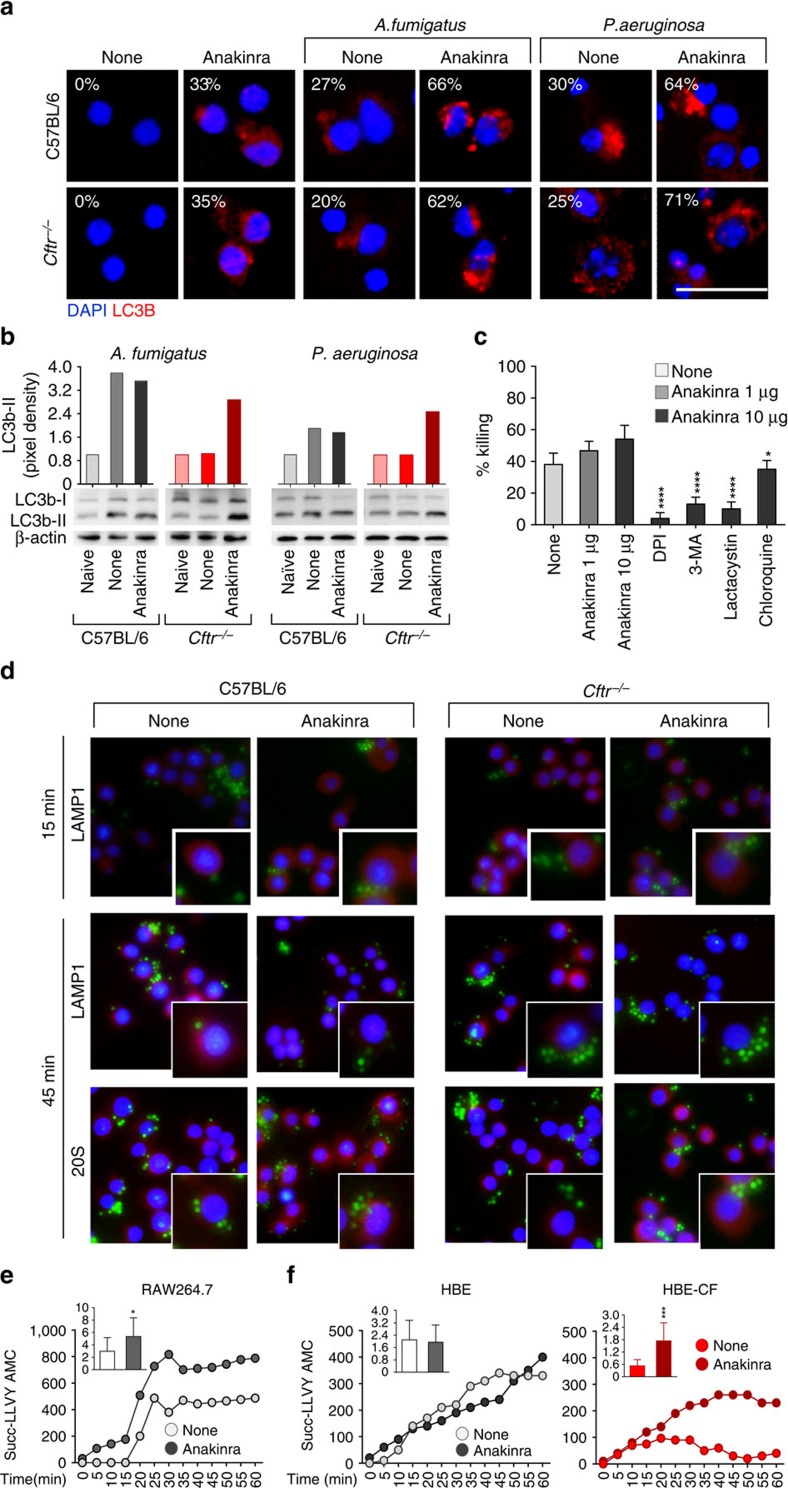
Anakinra promotes the autophagy and proteasomal degradation pathway. (**a**) Autophagy on purified lung macrophages from C57BL/6 or *Cftr*^*−/−*^mice stimulated with *Aspergillus* conidia or *P. aeruginosa* in the presence of 10 μg ml^−1^ anakinra and incubated with anti-LC3 antibody followed by PE secondary antibody. Representative images (original magnification, × 40) are shown. DAPI was used to detect nuclei. Numbers refer to % positive cells. Scale bar, 12.5 μm. (**b**) Density of bands from western blots of LC3b-I and II in homogenates of lungs of naive, *Aspergillus*- or *Pseudomonas*-infected untreated (none), or infected and anakinra-treated mice at 7 dpi. (**c**) Conidiocidal activity of RAW264.7 cells pretreated with DPI, 3-MA, lactacystin or chloroquine before the exposure to live *A. fumigatus* resting conidia for 2 h in the presence of anakinra. Conidiocidal activity refers to inhibition of CFUs (expressed as mean percentage±s.d. for all bar graphs, data pooled from three experiments). Each treatment had no effects on fungal growth in the absence of effector cells. *P<0.05, ****P<0.0001, pretreated versus anakinra 10 μg ml^−1^ only, one-way ANOVA, Bonferroni *post hoc* test. (**d**) Immunofluorescence imaging of purified alveolar macrophages from lungs of C57BL/6 or *Cftr*^*−/−*^mice after *in vitro* exposure to GFP-conidia at 37 °C for 2 h and chasing for 15 and 45 min. Formaldehyde-fixed cells were incubated with primary antibodies against Lamp1 or 20S followed by secondary anti-rabbit IgG–TRITC antibody. Nuclei were counterstained with DAPI. Images were acquired using EVOS FL Color Imaging System with × 40 objective. Shown are merged images of cells (a single cell is magnified in the inset) pulsed with GFP-conidia and red-stained for each compartment. Shown are representative data from three independent experiments.(**e**,**f**) RAW 247.3 (**e**) and HBE (**f**) cells from control or CF patients were stimulated with *Aspergillus* conidia in the presence of anakinra for 4 h and the amount of pmols of proteolytic activity was analysed with the proteasome activity assay kit (Abcam) Shown in the insets the relative slope with error bars representing the mean±s.d. *P<0.05, ***P<0.001, None versus anakinra, Student's *t*-test.

In this regard, autophagy is a mechanism involved in the intracellular defense against both *A. fumigatus*^38^,^39^ and *P. aeruginosa*^40^, is defective in CF^41^ and is induced by anakinra^13^. We monitored the effects of anakinra on autophagy induction in response to live conidia or bacteria in lung macrophages purified from C57BL/6 or *Cftr*^*–/–*^mice. Anakinra restored the defective autophagy in CF cells, as seen by LC3 immunofluorescence (Fig. 7a) and immunoblotting (Fig. 7b). Through autophagy, anakinra also increased the microbicidal activity of macrophages (Fig. 7c). Autophagy is activated by ROS and inhibited by chloroquine, a lysosomotropic agent that inhibits the fusion of autophagosome with lysosome and lysosomal protein degradation^42^. Accordingly, the anakinra activity was inhibited by chloroquine and the NADPH oxidase inhibitor diphenyleneiodonium DPI^43^, thus indicating that anakinra activates the autophagy/lysosomal degradation pathway. However, the anakinra activity was also inhibited by lactacystin, a known inhibitor of the proteasomal degradation pathway^44^. Thus, the activity of anakinra seems to rely on both the autophagy and the proteasome system, two cooperative and complementary degradation pathways^45^. Consistently, anakinra modified the intracellular routing of live conidia on phagocytosis. We followed the intracellular localization of green fluorescent protein (GFP)-conidia on lung macrophages purified from C57BL/6 and *Cftr*^*–/–*^mice by determining the colocalization and quantifying the degree of overlap with either the lysosomal-associated membrane protein 1 (LAMP1) or the 20S proteasome of protein degradation. Figure 7d shows that phagocytosed GFP-conidia rapidly (at 5 min) colocalized with LAMP1 in either type of macrophages. Colocalization was increased by anakinra. However, at 45 min, anakinra reduced the colocalization with LAMP1, while it increased the colocalization with the 20S proteasome (Fig. 7d). Consistent with the regulation of NLRP3 expression (Supplementary Fig. 8), the proteosomal degradation pathway in RAW cells (Fig. 7e) and in CF-HBE cells (Fig. 7f) was promoted by anakinra. Thus, anakinra not only controls the intracellular routing of pathogens upon phagocytosis through autophagy, but also reduces and NLRP3's half-life possibly via the proteasome system, and therefore controls both pathogen clearance and inflammation.

### Anakinra inhibits inflammasome activation in human CF

**Figure 8:**
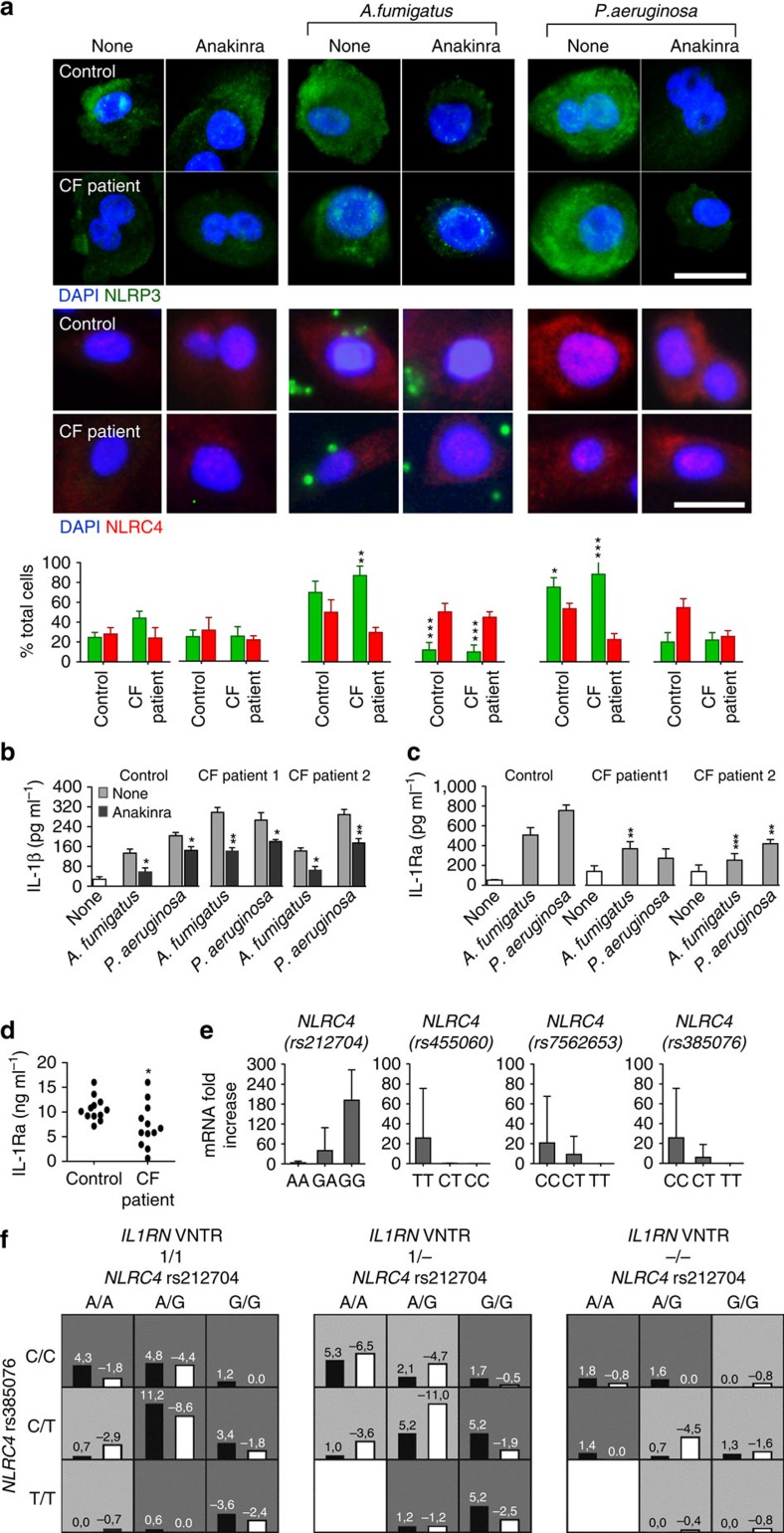
Anakinra inhibits inflammasome activation in human CF. (**a**) NLRP3 or NLRC4 staining of human bronchial epithelial (HBE) cells homozygous for ΔF508 mutation and control cells exposed to *P. aeruginosa* or *A. fumigatus* conidia at cells:microbes ratio of 2:1, and/or 10 μg ml^−1^ of anakinra. Images were acquired using the Olympus BX51 fluorescence microscope with a × 40 objective. Scale bar, 12.5 μm. DAPI was used to detect nuclei. Representative images of two independent experiments from three patients. Histograms indicate per cent of human bronchial epithelial cells with positive NLRP3 or NLRC4 expression. (**b**) IL-1β or (**c**) IL-1Ra in the supernatants of HBE cells from control or two CF patients exposed as above and (**d**) IL-1Ra levels in expectorates from control or CF patients (ELISA). (**e**) *NLRC4* expression by RT–PCR of CF patients carrying diverse genotypes at rs212704, rs455060, rs7562653 and rs385076. (**f**) Best three-factor model. Dark grey and light grey boxes correspond to the high- and low-risk genotype combinations, respectively. The left and right bars within each box correspond to *Pseudomonas*+ and *Pseudomonas*^–^, respectively. The top number above each bar is the sum of scores for the corresponding group of individuals. The heights of the bars are proportional to the sum of scores in each group. Data pooled from two experiments and presented as mean±s.d. for all bar graphs. *P<0.05, **P<0.01, ***P<0.001, treated *versus* untreated (none) (**a**,**b**) or control versus CF patient (**c**,**d**), Two-way ANOVA (**a**–**c**) Bonferroni *post hoc* test and two-sides Student's *t*-test (**d**).

To assess whether anakinra would also be able to inhibit NLRP3 activation in human CF bronchial ECs (CF-HBE), we evaluated NLRP3 and NLRC4 protein levels in primary HBE from non-CF patients and CF patients^46^ after 4 h of exposure to *A. fumigatus*, *P. aeruginosa* and/or anakinra. NLRP3, but not NLRC4, expression was increased in the presence of a pathogen in CF-HBE compared to control cells. Anakinra reduced the intensity of NLRP3 staining (Fig. 8a) and, concurrently, IL-1β production (Fig. 8b) without significantly affecting NLRC4 staining. Of interest, the levels of IL-1Ra were significantly lower in CF-HBE (Fig. 8c) and CF expectorates (Fig. 8d) as compared with controls, a finding indicating a defective NLRC4 activity in human CF.

### *NLRC4* and *IL1RN* polymorphisms affect microbial colonization

To assess whether genetic variants in the inflammasome/IL-1RI signalling pathway, and particularly genetic *NLRC4* deficiency, are risk factors for specific microbial colonization in CF, we evaluated nine gene variants (four in *NLRC4*, two in *NLRP3*, one in *IL1B* and one in *IL1RN*) in 284 CF patients (Supplementary Table 1). These variants were successfully genotyped in the samples (genotyping rate 94.3–100%), without displaying any deviation from the Hardy–Weinberg Equilibrium (Supplementary Table 2). Linkage disequilibrium (LD) analyses of the single-nucleotide polymorphisms (SNPs) in *NLRC4* and *NLRP3* (*r*^2^=0.03), failed to reveal the presence of significant LD blocks (Supplementary Fig. 10). For *A. fumigatus* colonization, significant associations were found for *NLRC4* ACTT haplotype (odds ratio (OR)=2.930, *P*=0.025) and for *NLRC4* rs212704 G/G genotype (OR=0.303, *P*=0.030; Table 1). To establish the functional effects of these SNPs we evaluated *NLRC4* expression in lung expectorates from genotyped CF patients. The AA genotype at rs212704, the CC genotype at rs455060, the TT genotype at the rs7562653 and the TT genotype at rs385076 show a reduction in *NLRC4* expression level compared with other genotypes (Fig. 8e). Therefore, genetic *NLRC4* deficiency, but not *NLRC4* sufficiency (rs212704), predisposes CF patients to *Aspergillus* colonization. No significant associations were found between *Aspergillus* colonization and the other genetic variants (Supplementary Tables 3–6) and no significant gene–gene interaction was detected (Supplementary Table 7). For *P. aeruginosa* colonization, no significant association was detected either at haplotype and single marker level (allele and genotype) tests (Supplementary Tables 8–11). However, on performing the generalized-multifactor dimensionality reduction analysis, two significant gene–gene interaction models (Table 1), involving *NLRC4* and *IL1RN* gene variants, significantly increased the risk of *Pseudomonas* colonization (*NLRC4* rs212704 × *IL1RN* VNTR, OR=3.018, Sign test *P*=0.001; *NLRC4* rs212704 × *NLRC4* rs385076 × *IL1RN* VNTR, OR=4.212, Sign test *P*=0.001; Fig. 8f). These data indicate that genetic deficiency of NLRC4, either alone or in combination with *IL-1R1N*, could be a risk factor for specific microbial colonization in the lungs of patients with CF.

## Discussion

The present study shows that the interplay between NLRP3 and NLRC4 governs host innate immune response and inflammation to colonizing microbes in murine and human CF. As infections have a negative impact on pulmonary functions in CF^47^, categorizing microbial colonization versus infection may help stratify CF patients for preventive treatment. NLRP3 activation contributed to neutrophil recruitment in both *A. fumigatus* and *P. aeruginosa* infections, a finding confirming the contribution of NLRP3, either alone^14^,^20^ or in association with other cytoplasmic sensors^24^, to the inflammatory responses in the lung. However, inflammation and tissue damage were greatly reduced in *Nlrp3*^*–/–*^ infected mice and NLRP3-dependent neutrophil infiltration and proinflammatory cytokine responses were associated with disease severity in conditions of unrestrained NLRP3 activity, such as in highly susceptible *Il1r8*^–/–^, *Il1ra*^–/–^ or *Nlrc4*^–/–^ mice. NLRP3 activity was instead defective in *Il1r1*^*–/–*^ mice, in which the attenuated IL-1β production was concomitant with a reduced disease severity during infections. While it is conceivable that the net functional activity of NLRP3 in the lung is also contingent, at least for *A*. *fumigatus*, on the fungal load (Supplementary Fig. 4), the fungal strain and route of infection^15^,^24^, our results indicate that the NLRP3 inflammasome is tightly regulated to avoid its aberrant activation^6^.

Indeed, NLRP3 was significantly increased in murine and human CF cells and its inhibition ameliorated inflammatory pathology in murine experimental infections and attenuated IL-1β production in human CF cells. The increased NLRP3 inflammatory response could be intrinsic to cells lacking CFTR due to the mitochondrial Ca^2+^ perturbation^20^. However, we found that NLRP3 activity could also be counteracted by the sustained production of IL-1Ra, to which NLRC4 greatly contributed. Several lines of evidence suggest an important function of NLRC4 for caspase-1 activation in response to intracellular as well as extracellular Gram-negative bacterial infection of the lung^17^,^18^,^21^,^27^. Activation of NLRC4 can be mediated by cytosolic bacterial flagellin^26^,^27^ and/or its cognate recognition by TLR5 (ref. 19), or be flagellin-independent through the type III secretion system^18^,^48^. In addition, activation of NLRC4 occurs after the assembly with members of the immune sensors NAIPs family^28^ that control ligand-dependent oligomerization of NLRC4 and may involve phosphorylation of the serine residue 553 by PKCδ (ref. 25). In pulmonary *P. aeruginosa* infection, NLRC4 activation occurs through the participation of members of the NAIPs family^28^ via an intact type III secretion system that mediates the cytosolic translocation of flagellin or PrgJ-like rod proteins^18^,^22^,^49^. In aspergillosis, we found that NLRC4 activation occurs through the TLR5/NAIP5/NF-κB-dependent pathway. Because both *Tlr5* and *Naip5* expressions failed to upregulate in the lungs of CF mice, decreased expression of TLR5 was observed in CF macrophages^50^, and *TLR5* gene variations influenced CF lung function^51^, these findings may account for the defective NLRC4 activation in CF. However, NLRC4 activation was also sensitive to calcium signalling. How the TLR5/NAIP5 and calcium signalling pathway specifically interact in NLRC4 activation and how it is affected by CFTR deficiency is unknown. However, it is intriguing that a Ca^2+^ flux occurs downstream TLR5 activation^52^ and tyrosine phosphorylation of PKCδ is calcium dependent^53^. Whatever the case, the increased [Ca^2+^]i and Ca^2+^-dependent signalling, known to promote NLRP3 activation^54^, actually restrains NLRC4 activation, a finding further pointing to the different regulation of either inflammasome in *Aspergillus* infection.

The defective NLRC4 activity observed in murine and human CF is a novel finding that may open new perspectives in the pathogenesis and therapy of inflammatory lung diseases in CF. *Nlrc4*^*–/–*^ mice produced more IL-1β and developed a more severe inflammatory response and tissue damage in both infections, despite their ability to restrict the pathogen growth, as already shown^21^. Disease susceptibility was dependent on NLRP3-driven inflammation, suggesting that NLRC4 may act as a negative regulator of NLRP3. Although the role of CFTR in myeloid cells is being appreciated^55^, we have found that defective NLRC4 activation in murine and human CF ECs resulted in an impaired production of IL-1Ra, which has the capacity to inhibit NLRP3 inflammasome activation. Although typically secreted by monocytes and PMNs, bioactive IL-1Ra isoforms could also be released from ECs in some conditions and may represent a mechanism by which IL-1 bioactivity is locally modulated^35^. However, as NLRC4 also regulates IL-18 production^56^, the low levels of IL-18 (this paper) and, consequently, of IFN-γ^3^ observed in CF could represent an additional mechanism by which NLRC4 regulates infections and inflammation in the respiratory tract.

Altogether, these findings point to the many similarities but also striking differences between NLRP3 and NLRC4 and add a layer of specificity to the innate immune response against the different pathogens that could be therapeutically exploited. A recent Cochrane review^57^ has provided no definitive conclusions on the safety and efficacy of the various immunosuppressive, anti-inflammatory drugs among patients with CF. Different from the anti-tumour necrosis factor approaches, associated with infectious complications, treatment with IL-1Ra has demonstrated an excellent safety profile, with no adverse reactions or superinfections with long-term treatment^58^. Anakinra decreased neutrophil infiltration, ameliorated tissue damage and inflammation against both *A. fumigatus* and *P. aeruginosa* infection, while decreasing NLRP3 activity in both murine and human CF. This occurred through the lysosomal pathway, through which pathogen clearance was promoted, and the ubiquitin/proteasomal degradation pathway through which NLRP3 activity was specifically controlled. Thus, anakinra appears to fulfill the requirement of an ideal immunomodulatory molecule capable of exerting pathogen control with minimum pathology. In addition, a recent report suggests that IL-1α, which is released when ECs become necrotic due to hypoxia, also significantly contributes to neutrophilic infiltration in the lungs of CF mice and this could be ameliorated by the use of anakinra^59^. IL-1α can induce IL-1β and results in an autoinflammatory loop that is also dependent on caspase-1 activation. Therefore, since anakinra will block both the effects of IL-1β and IL-1α and in addition has impact on several key inflammatory mechanisms such as NLRP3 inflammasome activation and autophagy, anakinra might be beneficial in CF by targeting multiple pathogenic mechanisms.

Finally, in line with the functional experimental data, the genetic analysis supports the role of *NLRC4* in determining the state of microbial colonization of CF patients. In particular, the significant association of the intronic SNP rs212704 in *NLRC4*, leading to defective *NLRC4* expression, with *A. fumigatus* colonization in CF patients suggests the presence of an ‘immunogenetic' background predisposing CF patients to specific microbial colonization. Moreover, consistent with the different activation of *NLRC4* by *Aspergillus* or *Pseudomona*s, the gene–gene interaction analyses clearly showed that *NLRC4* SNPs act synergistically with *IL1RN* VNTR^60^ to increase the susceptibility to *P. aeruginosa* colonization. Notably, the opposite (protective) allele IL1RN*2 has been previously associated with higher IL-1Ra and lower IL-1β release^60^.

In conclusion, our study shows that the elucidation of pathogenic, inflammasome-dependent mechanisms at the molecular level may help the development of novel strategies for patient stratification and personalized tailored approaches in human CF.

## Methods

### General experimental approaches

No samples, mice or data points were excluded from the reported analyses. No randomization procedure was implemented, however, mice were randomly assigned to group allocation at the time of purchase to minimize any potential bias. No blinding was applied on harvesting cells after the treatments.

### Mice

Six to 8-week female mice were used in all experiments. C57BL/6 mice were purchased from Charles River (Calco, Italy) and genetically engineered homozygote *Cftr*^*–/–*^ (*Cftr*^*tm1Unc*^) mice gut-corrected on C57BL/6 background^61^ were bred under specific pathogen-free conditions at the Animal Facility of San Raffaele Hospital, Milan, Italy. *Nlrp3*^*–/–*^, *Nlrc4*^*–/–*^ and *Il1ra*^*–/–*^ mice on the C57BL/6 background were bred under specific pathogen-free conditions at the Animal Facility of the University of Perugia, Perugia, Italy. Breeding pairs of *Nlrc4*^*–/–*^were obtained from Genetech (South San Francisco, CA, USA). Breeding pairs of *Nlrp3*^*–/–*^ were obtained from Dr Alessandra Mortellaro (Singapore Immunology Network, Agency of Science, Technology and Research, Biopolis, Singapore). *Il1r8*^*–/–*^and *Il1r1*^*–/–*^mice on the C57BL/6 background were bred under specific pathogen-free conditions at the Animal Facility at the Humanitas Hospital, Milan, Italy.

### Infections and treatments

Viable conidia (95%) of *A. fumigatus* (AF293) were obtained by growth on Sabouraud dextrose agar (Difco Laboratories, Detroit, MI, USA) supplemented with chloramphenicol for 5 days at room temperature. Mice were anaesthetized by intraperitoneal injection of 2.5% avertin (Sigma Chemical Co, St. Louis, MO) before intranasal instillation of a suspension of 2 × 10^7^ resting conidia per 20 μl saline and/or flagellin (Sigma-Aldrich) at 100 ng per 20 μl saline. For *P. aeruginosa* infection, clinical *P. aeruginosa* strain, isolated from a patient, was obtained from the Diagnostic Unit of Microbiology from the University of Perugia. The bacteria were grown for 3 h to reach exponential phase. Next, the bacteria were pelleted by centrifugation (2,700*g*, 15 min), washed twice with sterile PBS and the OD of the bacterial suspension was adjusted by spectrophotometry at 600 nm. The intended number of cfu was extrapolated from a standard growth curve. Appropriate dilutions with sterile PBS were made to prepare the inoculum before intranasal instillation of 3 × 10^7^ CFU per mice. Mice were monitored for fungal growth and histology. Microbial growth was expressed as log_10_ CFU per organ, mean±s.d. For histology, paraffin-embedded sections (3–4 μm) were stained with Periodic acid-Schiff (PAS). BAL fluid collection was done as described^3^. Differentials were determined by examination of cytocentrifuge slides after May Grunwald Giemsa staining (Sigma-Aldrich). Histology sections and cytospin preparations were observed using EVOS FL Color Imaging System (Fisher Scientific) and images were captured using high-sensitivity monochrome Sony ICX285AL CCD camera. Mice were treated intraperitoneally, from the day of infection until the end of the experiment, with anakinra 10 mg kg^−1^, daily, given the half-life of IL-1Ra of 6–8 h (ref. 62) or with 10 μg per mouse anti-IL1β antibody (RD systems, clone 30311) intranasally at the day of the infection and continuing every other day throughout the experiment.

### Cell preparation and culture

RAW264.7 cells (ATCC) were grown in RPMI 1640 medium supplemented with 1% Pen–Strep, 10 mM L-glutamine, 1% HEPES (Lonza, Basel Switzerland), 40 mg ml^−1^ gentamicin (Fisiopharma), 0,1% β-mercaptoethanol (Gibco, Thermo Scientific) and 10% FCS (Invitrogen, Life Technologies) at 37 °C in 5% CO_2_. Lung ECs were isolated from total lung cells by washing and resuspending lung fragments in prewarmed HBSS with 0.1 mg ml^−1^ DNase I and incubated at 37 °C for 1 h to allow enzymatic digestion. After blocking enzymatic activity with the addition of FBS the residues were magnetically separated with magnegtic beads (Miltenyi Biotech). Macrophages were isolated after 2 h plastic adherence at 37 °C. Neutrophils were positively selected with magnetic beads (Miltenyi Biotech) from the peritoneal cavity of uninfected mice 8 h after the intraperitoneal injection of 1 ml endotoxin-free 10% thioglycollate solution. Cells were plated in an 8-well culture slide or 12-well culture plate and stimulated for 4 h at 37 °C with 10 μg ml^−1^ flagellin, 10 μg ml^−1^ LPS (Sigma-Aldrich), live *Aspergillus* conidia or *P. aeruginosa* at the ratio cells:microbes (1:1) or fungal antigens (10 μg ml^−1^)^63^. ATP (Sigma-Aldrich), 2 mM, was added 30 min before the end of the experiment. EDTA 2 mM was added at the same time as the infection. Cells were then fixed with 4% paraformaldyde and stained with anti-NLRP3 (Cat. Number ab4207, Abcam) followed by goat anti-mouse Alexa Fluor 555 (Clone Poly4053, Biolegend) or anti-pNLRC4 (Genetech) followed by polyclonal anti-hamster FITC (Sigma-Aldrich) and Ipaf (Cat. Number 06-1125, Millipore) followed by polyclonal anti-rabbit TRITC (Sigma-Aldrich). All primary antibodies were used at the concentration of 0.5 μg ml^−1^ and secondary TRICT or FITC coniugated antibodies at 1 μg ml^−1^. Images were acquired using a fluorescence microscope (BX51 Olympus) with a × 20 objective and the analySIS image processing software (Olympus). 4′-6-Diamino-2-phenylindole (DAPI, Molecular Probes, Invitrogen Srl., Milan, Italy) was used to counterstain tissues and to detect nuclei. A minimum of 100 cells were counted per group and the percentages indicating the number of cells showing either NLRP3 or PNLRC4 localization was obtained. Cell extracts were analysed by western blotting and supernatants for IL-1Ra content by ELISA. For NLRP3 half-life experiments, cycloheximide, 40 μg ml^−1^, treatment was carried out, with or without 10 μg ml^−1^ anakinra, at various time points in 0% FBS medium, on RAW264.7 cells stimulated with LPS (10 μg ml^−1^) for its ability to increase NLRP3 half-life^37^ and/or MG132 (20 μg ml^−1^) or leupeptin (20 μg ml^−1^). Cell lysates were assessed for NLRP3 protein expression by western blotting.

### siRNA design and delivery

The Integrated DNA technologies-pool duplexes of predesigned siRNA (*Nlrp3*, dupex name: MMC.RNAI.N010552.12.1, *Nlrc4* dupex name: MMC.RNAI.N010552.12.1, *Tlr5* duplex name: (MMC.RNAI.N016928.12.1), *Naip2* duplex name: (MMC.RNAI.N010872.12.1) and *Naip5* duplex name: (MMC.RNAI.N010870.12.1) were purchased from IDT (TEMA ricerca, Italy). For *in vivo* studies, each mouse was lightly anaesthetized by inhaled diethyl ether, then given intranasal administration of unmodified siRNA (10 mg kg^−1^) or equivalent doses of nonspecific control siRNA duplex in a volume of 20 μl of duplex buffer (IDT, Tema ricerca). It is known that lung-specific siRNA delivery can be achieved by intranasal administration without the need for viral vectors or transfection agents *in vivo*^3^. Intranasal siRNA was given twice 2 days before infection or 3 days after infection. For *in vitro* studies, specific predesigned siRNA was transfected into RAW264.7 macrophages using TransIT-TKO Transfection Reagent (Mirus) and incubated for 24 h (as indicated by preliminary experiments performed at 12, 24 or 48 h) at 37 °C in 5% CO_2_. The efficiency of gene silencing was assessed by RT–PCR at 48 h in unstimulated cells (Supplementary Fig. 11). Cells were pretreated with the 100 μM NF-κB inhibitor, SN50 (Calbiochem) for 30 min.

### Immunofluorescence and immunohistochemistry

The lung was removed and fixed in 10% phosphate-buffered formalin, embedded in paraffin and sectioned at 5 μm. Sections were then rehydrated and after antigen retrieval in citrate buffer (10 mM, pH6), sections were fixed in 2% formaldehyde for 40 min at room temperature and permeabilized in a blocking buffer containing 5% FBS, 3% BSA, and 0.5% Triton X-100 in PBS. The slides were then incubated at 4 °C with primary antibodies anti-NLRP3 (Cat. Number ab4207, Abcam) or anti-pNLRC4 (Genetech) and anti-NLRC4 (Cat. Number 06-1125, Millipore). After extensive washing with PBS, the slides were then incubated at room temperature for 60 min with secondary antibodies, for NLRP3 goat anti-mouse Alexa Fluor 555 (Clone Poly4053 Biolegend) for pNLRC4 polyclonal anti-hamster FITC (Sigma-Aldrich) and for NLRP4, anti-rabbit TRITC (Sigma-Aldrich). Nuclei were counterstained with DAPI. Corresponding average immunofluorescence intensities of NLRP3 and NLRC4 were calculated by using Image J software calculated on 142 × 142 pixel area^64^. For immunohistochemistry, sections were incubated overnight with anti-NLRP3 (Abcam) or polyclonal NLRC4 (Millipore) followed by biotinylated secondary antibodies. Cells were counterstained with hematoxylin or DAPI to detect nuclei. For intracellular routing of conidia, purified alveolar macrophages were plated in complete medium into chambered coverglass (Lab-Tek/Nunc; Thermo Scientific) in a temperature-regulated environmental chamber and exposed to GFP-conidia (at a 1:4 cell/conidia ratio), in serum free RPMI-1640 medium and time chased at 45 min. After washout, cells were fixed, permeabilized and incubated at 4 °C with primary antibodies produced in rabbit against LAMP1 or 20S (Sigma-Aldrich) as described^65^. After extensive washing with PBS, the cells were incubated at room temperature for 60 min with 1:400 secondary anti-rabbit IgG–TRITC antibody (Sigma-Aldrich). All images were acquired using a fluorescence microscope. BX51 Olympus with a × 20, × 40 and × 100 objective with the analySIS image processing software (Olympus) or EVOS FL Color Imaging System with a × 40 and × 60 objective.

### Terminal deoxynucleotidyl transferase-mediated deoxyuridine triphosphate nick-end labelling of lung sections

The lungs were fixed in 4% buffered paraformaldehyde, pH 7.3, for 36 h and embedded in paraffin. Sections were deparaffinized, rehydrated and treated with 0.1 M citrate buffer, pH 6.0, for 20 min in a water bath, washed and blocked in 0.1 M Tris/HCl buffer, pH 7.5, supplemented with 3% bovine serum albumin and 20% FCS. The slides were then incubated with fluorescein-coupled dUTP and TUNEL enzyme (Roche Diagnostics) in the presence of terminal deoxynucleotidyl transferase. The samples were then washed with PBS, incubated for 10 min at 70 °C to remove unspecific binding. The sections were mounted and analysed by fluorescent microscopy using a × 40 objective.

### Autophagy

For autophagy on lung cells, 1 × 10^6^ purified alveolar macrophages were stimulated on glass slides in 24 multiwell plates with *A. fumigatus* live conidia or *P. aeuginosa* and incubated for two at 37 °C in 5% CO_2_. Cells were incubated with 1:200 diluted anti-LC3 antibody (Cell Signaling Technology) overnight at 4 °C in PBS containing 3% normal bovine serum albumin, incubated with anti-rabbit–PE secondary antibody (Sigma-Aldrich), and fixed for 20 min in PBS containing 4% paraformaldehyde. Images were acquired using the Olympus BX51 fluorescence microscope with a × 40 objective and analySIS image processing software (Olympus). DAPI was used to detect nuclei. An equal amount of cell lysate in 2 × Laemmli buffer (Sigma-Aldrich) was probed with rabbit anti-LC3b-I or anti-LC3b-II antibody (Abcam) for immunoblotting.

### Proteasome assay

For studying proteasome activity, cells were plated on a 12-well cell culture plate and stimulated with *Aspergillus* conidia in the presence of anakinra for 4 h at 37 °C. After incubation cells were lysed with NP-40 lysis buffer and the amount of pmols of proteolytic activity was analysed with the proteasome activity assay kit (Abcam) that takes advantage of the chymotrypsin-like activity, utilizing an AMC-tagged peptide substrate (Proteasome Substrate (Succ-LLVY-AMC in DMSO)), which releases free, highly fluorescent AMC in the presence of proteolytic activity.

### Conidiocidal activity

RAW264.7 cells were plated on 96-well cell culture plate in supplemented RPMI medium. Cells were pretreatment for 30 min with either 2.5 μM chloroquine (an acidophilic weak base that inhibits endosomal acidification), 5 μg ml^−1^ brefeldin A (to inhibit the vacuolar pathway), 50 μM lactacystin (to inhibit proteasomal degradation), 5 mM 3-MA (to inhibit autophagy), 2 μg ml^−1^ DPI (to inhibit reactive oxygen species generation) and 50 μM rapamycin (to induce autophagy), exposed for 2 h to10 μg ml^−1^ of anakinra and stimulated with live *A. fumigatus* resting conidia for 2 h (10:1 cells/fungus ratio). Inhibition of CFUs (expressed as mean percentage±s.d.), referred to as conidiocidal activity, was determined as described^63^.

### Human bronchial epithelial cells

HBE cells, homozygous for the ΔF508 mutation and its isogenic wild type were obtained from lung transplants (CF patients) or lung resections (non-CF patients) from and cultured as described^46^. Cells were maintained at 37 °C in a humidified incubator in an atmosphere containing 5% CO_2_, and the experiments were done 5 days after plating. Cells were exposed to *A. fumigatus* conidia or *P. aeruginosa* at cells:microbes ratio of 2:1, and treated with 10 μg ml^−1^ anakinra or vehicle. Cells were incubated for 4 h at 37 °C in 5% CO_2_ (as indicated by preliminary experiments). Cultures growing on culture slides were fixed for 20 min in PBS containing 4% paraformaldehyde then incubated with human anti-NLRP3 at 4 °C in PBS containing 3% normal bovine serum albumin, washed and incubated with anti-mouse-TRITC secondary antibody (Sigma-Aldrich). Images were acquired using the Olympus BX51 fluorescence microscope with a × 40 objective and the analySIS image processing software (Olympus). DAPI was used to detect nuclei.

### Reverse transcriptase-PCR and ELISA

The levels of cytokines in lung homogenates and supernatants were determined by ELISAs (R&D Systems). Real-time RT–PCR was performed using the BioRad CFX96 System and SYBR Green chemistry (BioRad). Cells were lysed and total RNA was reverse transcribed with cDNA Synthesis Kit (BioRad), according to the manufacturer's instructions. Amplification efficiencies were validated and normalized against GAPDH. The thermal profile for SYBR Green real-time PCR was at 95 °C for 3 min, followed by 40 cycles of denaturation for 30 s at 95 °C and an annealing/extension step of 30 s at 60 °C. Each data point was examined for integrity by analysis of the amplification plot. The messenger RNA (mRNA)-normalized data were expressed as relative gene mRNA in treated compared with untreated experimental groups or cells.

### Western blotting

Blots of cell lysates were incubated with antibodies against the following proteins: Caspase-1p10 (M-20 Cat. Number sc-514, Santa Cruz Biotechnology), NLRP3 (Cat. Number ab4207, Abcam), NLRC4 (Cat. Number 06-1125, Millipore) and pNLRC4 (Genetech), LC3b-I or LC3b-II (Abcam) followed by IgG–HRP-conjugated secondary antibody (Sigma–Aldrich) after separation in 12% Tris/glycine SDS gel and transfer to a nitrocellulose membrane. Normalization was performed probing the membrane with mouse-anti-β-actin antibody (Sigma–Aldrich). Chemiluminescence detection was performed with LiteAblotPlus chemiluminescence substrate (Euroclone S.p.A), using the ChemiDocTM XRS+Imaging system (Bio-Rad), and quantification was obtained by densitometry image analysis using Image Lab 3.1.1 software (Bio-Rad). Images have been cropped for presentation. Full size images are presented in Supplementary Figs 12-14.

### Human studies

*Patient data*. A prospective multicenter longitudinal genetic association study involving 284 patients of Caucasian origin who had a proven diagnosis of CF (*CFTR* genotyping, sweat testing and clinical phenotype) was performed (Supplementary Table 1). Clinical records from each patient were reviewed and clinical data including age, gender, lung function testing, measures of nutrition, microbiological findings and vital status were abstracted. *A. fumigatus* or *P. aeruginosa* positivity was defined as the presence of persistent positive *Aspergillus* cultures, but negative galactomannan and no immunological responses or persistent, for at least 6 months, *Pseudomonas*, respectively.

*SNPs selection and genotyping*. Patients provided a blood specimen for DNA isolation performed using the QIAamp DNA Mini (Qiagen, Milan, Italy) following the manufacturer's instructions and stored at −20 °C. Supplementary Table 1 SNPs in *NLRC4* and *NLRP3* were selected based on their ability to tag surrounding variants in the HapMap-CEU population of the International HapMap project, NCBI build B36 assembly HapMap phase III (http://www.hapmap.org). Haplotype-based tagging SNPs were selected by assessing LD blocks from the genes of interest with a pairwise correlation coefficient *r*^2^ of at least 0.80 and a minor allele frequency higher than 5% in the HapMap-CEU population. In addition, the rs1143627 SNP in *IL1B* and the 1,2 variable number of tandem repeats (VNTRs) in *IL1RN* were also studied. SNP genotyping was performed by KASPar assays (KBiosciences, Hertfordshire, UK) according to manufacturer's instructions using Applied Biosystems 7500 Fast qPCR system (Life Technologies, Milan, Italy). The *IL1RN* VNTR polymorphism (86-bp repeat in intron 2) was analysed as previously described^66^. The PCR products were analysed by 2% agarose gel electrophoresis. Genotyping sets comprised randomly selected replicates of previously typed samples and two negative controls (water). Concordant genotyping was obtained for ≥99%.

*Statistical analysis*. Data are expressed as mean±s.d. Horizontal bars indicate the means. Statistical significance was calculated by one or two-way ANOVA (Bonferroni's *post hoc* test) for multiple comparisons and by a two tailed Student's *t*-test for single comparison. Since the distribution of levels tested by Kolmogorov–Smirnov normality test turned out to be non-significant. We considered all *P* values ≤0.05 significant. The data reported are either representative from three representative experiments (histology, immunofluoresce, TUNEL and western blotting) or pooled otherwise. The *in vivo* groups consisted of 4–6 mice per group. Data were analysed by GraphPad Prism 4.03 program (GraphPad Software). No statistical method was used to predetermine sample size. For human data, Hardy–Weinberg equilibrium (HWE) was tested using Haploview v4.2. LD analysis was performed using Haploview, and defining LD blocks based on the solid spine of LD algorithm^67^. Case–control single marker and haplotype association tests were performed using UNPHASED^68^ under an additive model and adjusting for sex and age at sampling. Gene–gene interaction analyses were performed with the Generalized-Multifactor Dimensionality Reduction method (version 0.9)^69^. This method uses the same data-reduction strategy as the original MDR method^70^, but score-based statistics using maximum-likelihood estimates are introduced to classify multifactor cells into two different groups^69^. As in MDR, the best *n*-locus model is selected based on the highest training accuracy, and the maximum balanced testing accuracy and cross validation consistency are used to determine the overall best epistasis model. Balanced testing accuracy is calculated using the formula (sensitivity+specificity)/2 to yield an unbiased estimate for unbalanced case–control studies. Last, the *χ*^2^ and sign tests determine whether the factors are significantly associated with the phenotype of interest. In particular, a significant *χ*^2^-test reveals that the SNP or interaction between SNPs is associated with phenotype tested, while a significant sign test suggests that the best model with one or more SNPs is significantly better than the null model. Two-tail *P* values are reported. Bonferroni's correction for multiple testing was not performed since we are assessing specific questions on genes involved in functional pathways related to *Pseudomonas* and *Aspergillus* infection and we are not searching for associations without *a priori* hypotheses^71^.

### Study approval

Murine experiments were performed according to the Italian Approved Animal Welfare Authorization 360/2015-PR and Legislative decree 26/2014 regarding the animal licence obtained by the Italian Ministry of Health lasting for five years (2015–2020). Infections were performed under avertin anaesthesia, and all efforts were made to minimize suffering. Human studies approval was obtained from institutional review boards at each site and written informed consent was obtained from the participants, or, in case of minors, from parents or guardian.

## Additional information

**How to cite this article:** Iannitti, R. G. *et al.* IL-1 receptor antagonist ameliorates inflammasome-dependent inflammation in murine and human Cystic Fibrosis. *Nat. Commun.* 7:10791 doi: 10.1038/ncomms10791 (2016).

## Supplementary Material

Supplementary Information Supplementary Figures 1-14, Supplementary Tables 1-11 and Supplementary References

## Figures and Tables

**Table 1:** Haplotype (a) and genotype (b)* association study between *NLRC4* and *Aspergillus* infection; (c)† best models assessed by the GMDR for one to five-way combinations, to test gene–gene interactions in determining *Pseudomonas* infection.

(a)								
***NLRC4*****Haplotype**‡	***NLRC4*** **SNPs**				**Asp−**	**Asp+**	**OR**	***P*** **value**
	**rs212704**	**rs455060**	**rs7562653**	**rs385076**				
H1	A	T	C	C	122 (40.8%)	34 (47.6%)	Reference	
H2	G	C	T	T	72 (24.1%)	20 (28.0%)	0.875	0.729
H3	G	T	C	C	39 (13.1%)	8 (10.8%)	0.812	0.644
H4	G	C	C	T	32 (10.7%)	1 (1.4%)	0.169	0.177
H5	G	C	C	C	11 (3.8%)	1 (1.4%)	0.387	0.452
H6	A	T	T	C	8 (2.7%)	—	0.000	0.268
**H7**	**A**	**C**	**T**	**T**	**5 (1.7%)**	**4 (5.3%)**	**2.930**	**0.025**
H8	A	T	T	T	4 (1.4%)	1 (1.4%)	0.854	0.995
H9	A	T	C	T	1 (0.4%)	1 (1.4%)	4.879	0.234
H10	G	C	T	C	—	1 (1.4%)	9.989e+013	0.149

(b)						
**Status**	***NLRC4*** **rs212704**			**A/G OR (*****P*****-value)**	**G/G OR (*****P*****-value)**	**Global** ***P*****-value**
	**A/A (%)**	**A/G (%)**	**G/G (%)**			
Asp−	39 (22.9%)	83 (48.8%)	48 (28.2%)	0.996 (0.174)	**0.303 (0.030)**	0.071
Asp+	9 (25.0%)	23 (63.9%)	4 (11.1%)			
	***NLRC4*** **rs455060**			**C/T OR (*****P*****-value)**	**T/T OR (*****P*****-value)**	**Global** ***P*****-value**
**Status**	**C/C (%)**	**C/T (%)**	**T/T (%)**			
Asp−	20 (12.2%)	90 (54.9%)	54 (32.9%)	0.750 (0.778)	0.774 (0.951)	0.881
Asp +	6 (15.4%)	21 (53.9%)	12 (30.8%)			
	***NLRC4*** **rs7562653**			**C/T OR (*****P*****-value)**	**T/T OR (*****P*****-value)**	**Global** ***P*****-value**
**Status**	**C/C (%)**	**C/T (%)**	**T/T (%)**			
Asp−	83 (46.6%)	79 (44.4%)	16 (9.0%)	1.161 (0.691)	1.009 (0.900)	0.924
Asp +	15 (37.5%)	21 (52.5%)	4 (10.0%)			
	***NLRC4*** **rs385076**			**C/T OR (*****P*****-value)**	**T/T OR (*****P*****-value)**	**Global** ***P*****-value**
**Status**	**C/C (%)**	**C/T (%)**	**T/T (%)**			
Asp−	61 (35.1%)	86 (49.4%)	27 (15.5%)	1.334 (0.260)	0.643 (0.274)	0.415
Asp +	11 (28.2%)	24 (61.5%)	4 (10.3%)			

(c)				
**Model**	**TBA**	**Sign Test (P)**	**CVC**	**OR (95% C.I.)**
*NLRC4 rs212704*	0.516	6 (0.377)	9/10	2.150 (0.955-4.841)
***NLRC4 rs212704*** × ***IL1RN VNTR***	**0.607**	**10 (0.001)**	**10/10**	**3.018 (1.464-6.219)**
***NLRC4 rs212704*** × ***NLRC4 rs385076*** × ***IL1RN VNTR***	**0.612**	**10 (0.001)**	**10/10**	**4.212 (1.981-8.957)**
*NLRC4 rs212704* × *NLRP3 rs10925026 x IL1RN VNTR* × *IL1B rs1143627*	0.530	7 (0.172)	6/10	6.024 (2.795-12.986)
*NLRC4 rs212704* × *NLRC4 rs7562653* × *NLRP3 rs10925026* × *IL1RN VNTR* × *IL1B rs1143627*	0.519	6 (0.377)	9/10	12.622 (5.429-29.346)

SNP, single-nucleotide polymorphism; CVC, cross-validation consistency; TBA, testing balanced accuracy.

^*^Association tests were carried out using UNPHASED^41^ adjusting for sex and age at sampling. Significant results are shown in bold.

^†^Significant interaction models are shown in bold.

^‡^Global haplotype test, *P* value=0.092.
