# Supplementary material for: IL-1 receptor antagonist ameliorates inflammasome-dependent inflammation in murine and human cystic fibrosis
Source: Nat Commun. 2016 Mar 14;7:10791. doi: 10.1038/ncomms10791 (PMC4793079; doi:10.1038/ncomms10791)
Supplement: Supplementary Information — Supplementary Figures 1-14, Supplementary Tables 1-11 and Supplementary References [file ncomms10791-s1.pdf]

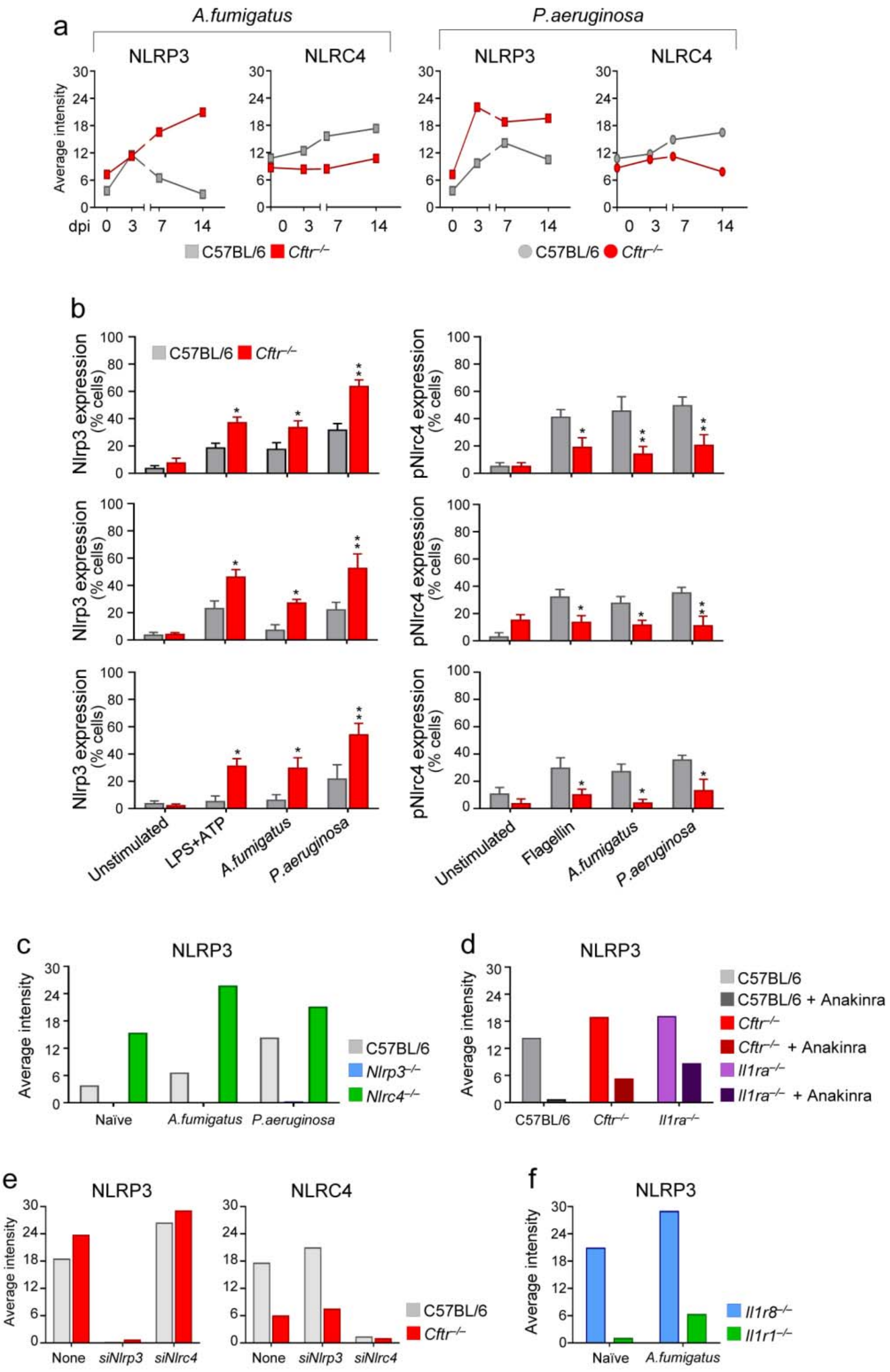

Supplementary Figure 1. Quantification of the corresponding average immunofluorescence intensities of NLRP3 and NLRC4 protein expression relative to (**a**) Fig. 1e and f, (**c**) Fig. 5b, (**d**) Fig. 6f and m, (**e**) Supplementary Fig. 5d and (**f**) Supplementary Fig. 6. Average intensities were calculated by using Image J software calculated on 142x142 pixel area. (**b**) Number of cells with positive NLRP3 or NLRC4 expression staining relative to Figure 2c and d. A minimum of 100 cells were counted per group. *P<0.05, **P<0.01, stimulated *vs* unstimulated cells, Two-way ANOVA Bonferroni post test.

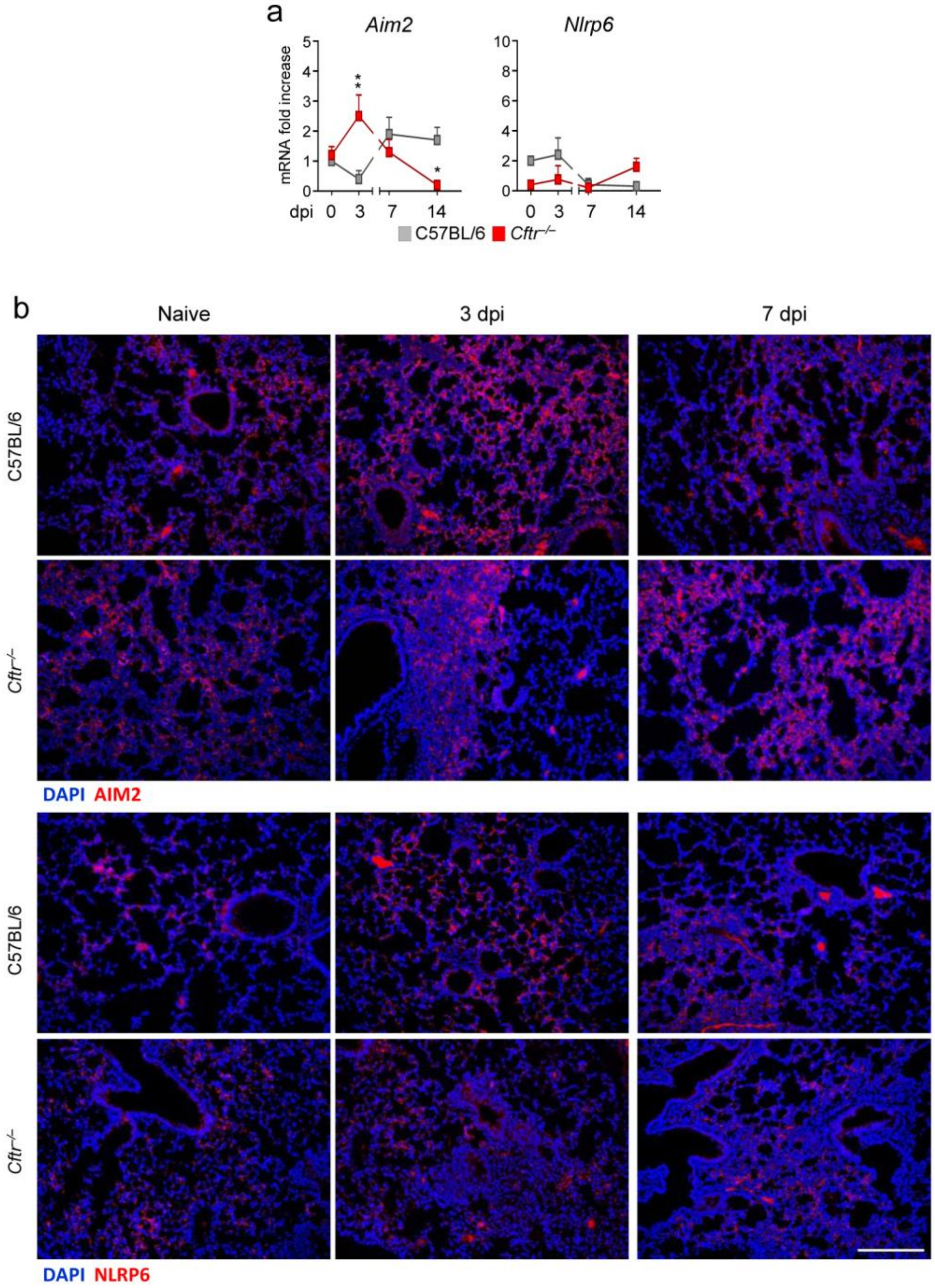

Supplementary Figure 2. Aim2 and NLRP6 expression in murine CF. C57BL/6 and *Cftr*$^{-/-}$ mice (*n*=6 for all groups) were infected intranasally with live *A.fumigatus* conidia and assessed for AIM2 and NLRP6 expression in the lungs at different days post-infection (dpi) by RT-PCR and immunofluorescence staining with polyclonal rabbit anti-AIM2 or NLRP6 antibody followed by anti-rabbit TRICT. (**e**) Representative images of two independent experiments were acquired with a ×20 objective. Scale bars, 200 µm, insert 50 µm. *P<0.05, **P<0.01, C57BL/6 *vs* *Cftr*$^{-/-}$ mice at different dpi, Two-way ANOVA Bonferroni post test.

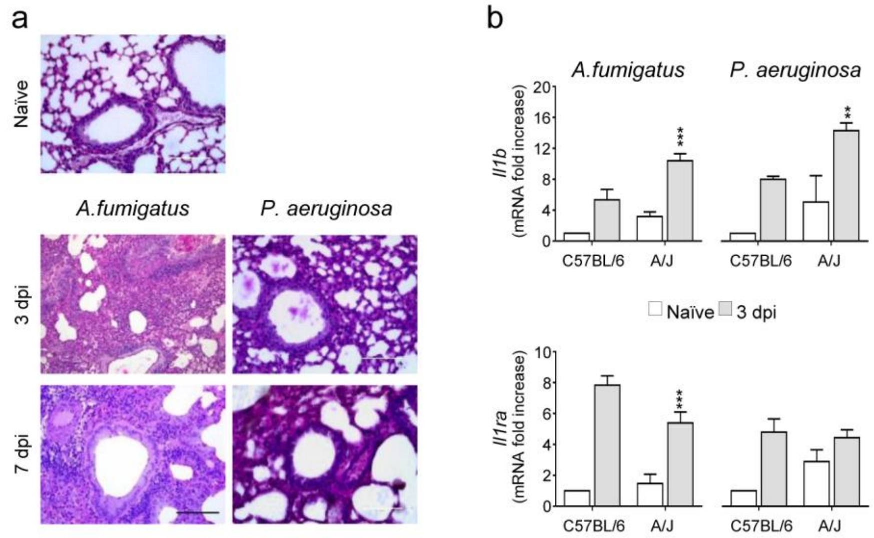

Supplementary Figure 3. Susceptibility of A/J mice to *A.fumigatus* or *P.aeruginosa* infection. A/J mice (*n*=6 for all groups) were infected intranasally with live *A.fumigatus* conidia or *P.aeruginosa* and assessed for (**a**) lung inflammatory pathology at different days-post-infection (dpi) and (**b**) *Il1b* and *Il1ra* gene expression in total lung cells at 7 dpi by RT-PCR. Representative images of two independent experiments were acquired using EVOS® FL Color Imaging System with a ×40 objective. Scale bar 100 µm. Data pooled from two experiments and presented as mean ± SD for all bar graphs. **P<0.01, ***P<0.001, C57BL/6 *vs* A/J mice at different dpi, Two-way ANOVA Bonferroni post test.

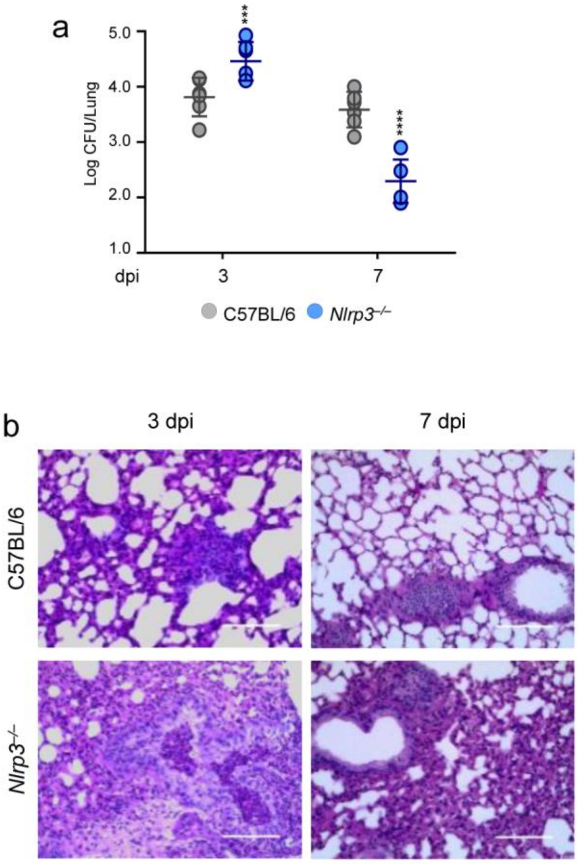

Supplementary Figure 4. Susceptibility of *Nlrp3*$^{-/-}$ mice to 8x10^{9} *A.fumigatus* conidia. C57BL/6 or *Nlrp3*$^{-/-}$ mice (*n*=6 for all groups) were infected intranasally with 8x10^{9}/live *A.fumigatus* conidia and accessed for (**a**) fungal growth (log CFU, mean ±SD) and (**b**) lung histology at 3 and 7 dpi (periodic acid-Schiff staining). Representative images of two independent experiments were acquired using EVOS® FL Color Imaging System with a ×40 objective. Scale bar 100 µm. Data pooled from two experiments and presented as mean ± SD for all bar graphs. ***P<0.001, C57BL/6 *vs Nlrp3*$^{-/-}$ mice at different dpi, Two-way ANOVA Bonferroni post test.

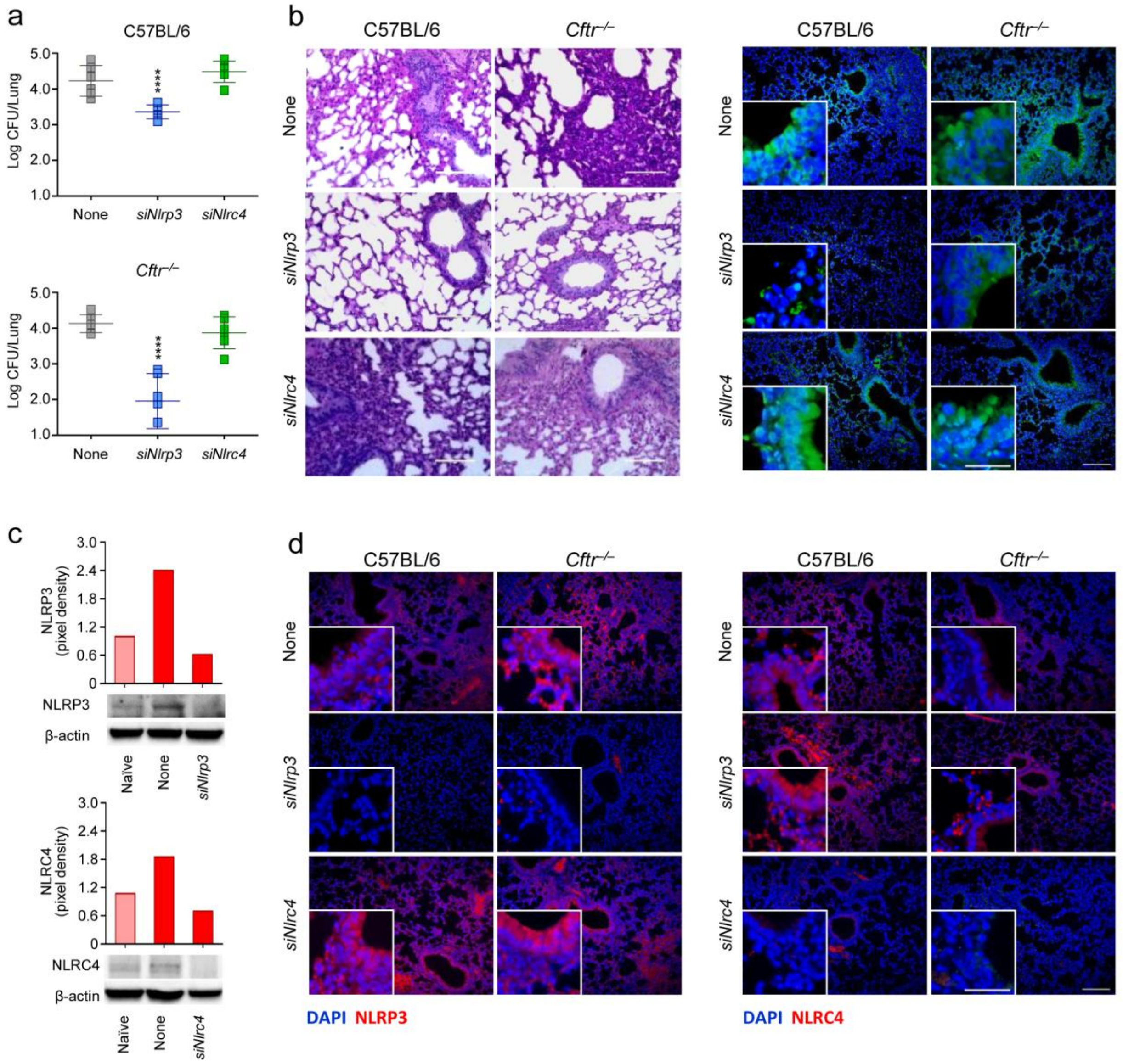

Supplementary Figure 5. Susceptibility to *P.aeruginosa* infection of mice treated with *siNlrp3* or *siNlrc4*. C57BL/6 or *Cftr*$^{-/-}$ mice (*n*=6 for all groups) were infected intranasally with live *P.aeruginosa* and treated with specific *Nlrp3* or *Nlrc4* siRNA or scrambled siRNA (siScram) and assessed for (**a**) bacterial growth (log CFU, mean ± SD) in the lungs at 7dpi; (**b**) lung histology (periodic acid-Schiff staining) and increased deposition of DNA on lung parenchyma cells on TUNEL staining. Cell nuclei were stained blue with DAPI. (**c**) NLRP3 and NLRC4 protein expression in lung immunoblots of infected and siRNA treated *Cftr*$^{-/-}$ mice at 7 dpi. (**d**) Immunofluorescence staining of NLRP3 in the lungs of infected and above treated mice. Cell nuclei were stained blue with DAPI. Representative images of two independent experiments were acquired using EVOS® FL Color Imaging System with a ×40 objective for histology (Scale bar 100 μm) and a high-resolution Microscopy Olympus DP71 using a ×20 objective for TUNEL and immunofluorescence (Scale bars 200 μm, insert 50 μm). Data pooled from two experiments and presented as mean $\pm$ SD for all bar graphs. ***P<0.001, untreated (none) *vs* siRNA treated mice, One-way ANOVA Bonferroni post test. For NLRP3 or NLRC4 quantification, see Supplementary Fig. 1.

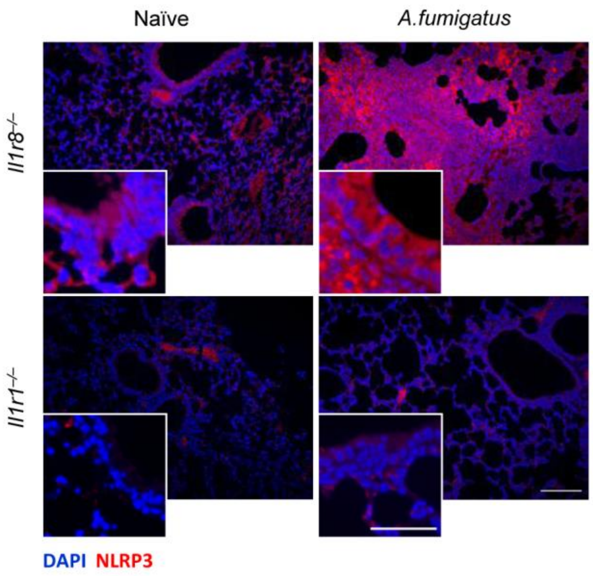

Supplementary Figure 6. NLRP3 and NLRC4 expression in lungs of *Il1r8*$^{-/-}$ or *Il1r1*$^{-/-}$ mice. Immunofluorescence staining of NLRP3 in the lungs of naïve or *A.fumigatus* conidia infected *Il1r8*$^{-/-}$ and *Il1r1*$^{-/-}$ infected mice at 7 dpi. DAPI was used to detect nuclei. Representative images of two independent experiments. Photographs were taken using a high-resolution Microscopy Olympus DP71 using a ×20 objective. Scale bars 200 μm, insert 50 μm. For NLRP3 quantification, see Supplementary Fig. 1.

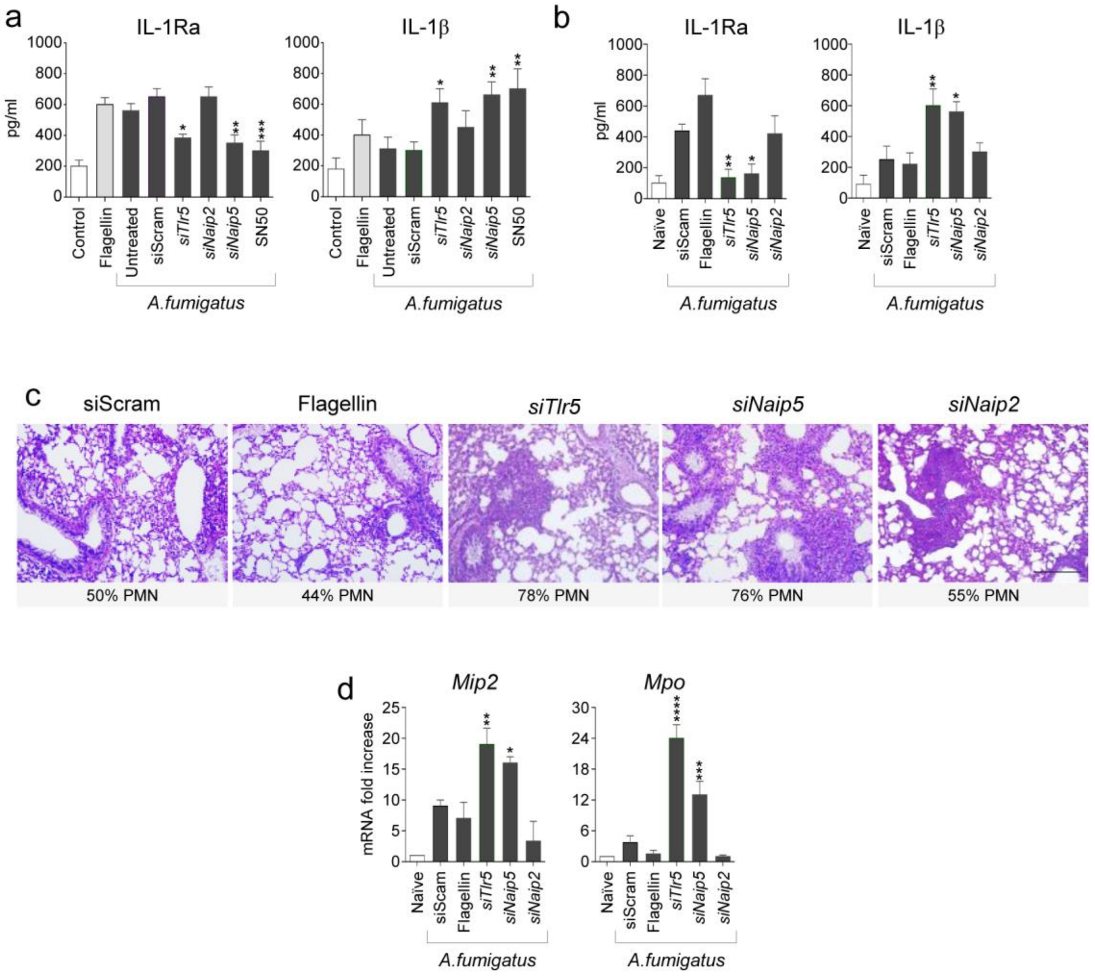

Supplementary Figure 7. The TLR5/NAIP5/NLRC4 pathway regulates inflammation. (**a**) Cells were pre-treated with specific siRNA for *Tlr5*, *Naip2*, *Naip5* or scrambled siRNA (siScram) or the NF-κB inhibitor SN50, and exposed to flagellin or *Aspergillus* conidia for 2 h at 37°C before the assessment of cytokine levels (ELISA) in the supernatants. C57BL/6 mice were given siRNA intranasally twice, two days before and 3 days after the infection before the assessment of (**b**) cytokine levels in lung homogenates, (**c**) lung histology (periodic acid-Schiff staining) and (**d**) *Mip2* and *Mpo* gene expression by RT-PCR on total lung cells at 4 dpi. Representative images of 3 independent experiments were acquired using EVOS® FL Color Imaging System with a ×40 objective. Scale bar 100 µm. Data pooled from two experiments and presented as mean ± SD for all bar graphs. *P<0.05, **P<0.01, untreated *vs* treated mice, one-way ANOVA Bonferroni post test.

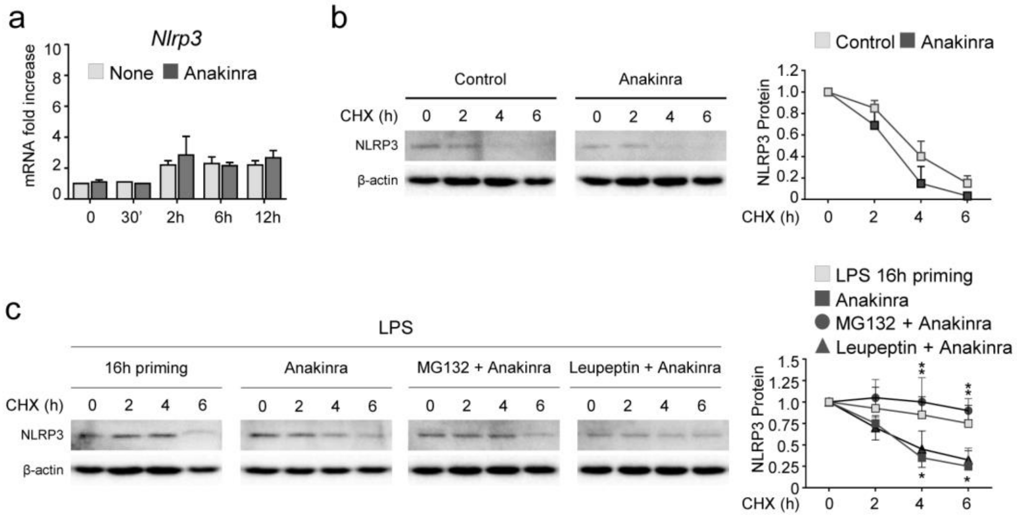

Supplementary Figure 8. Anakirna promotes NLRP3 degradation via the proteasome system. (**a**) Messanger RNA expression-fold increase of NLRP3 in LPS-primed (for the indicated period of time) RAW264.7 cells in the presence of 10 µg/ml of anakinra showing no increase in mRNA expression by anakinra. (**b** and **c**) Cells were incubated with CHX (40 µg/ml, to block new protein synthesis) for various times without (**b**) or with (**c**) 16 h priming with LPS in the presence of anakinra, the proteasomal inhibitor MG132 (40 µg/ml) or the lysosomal inhibitor leupeptin (20 µg/ml) before immunoblotting for NLRP3 and densitometric plot analysis of NLRP3 decay *vs* time of CHX exposure with a best fit line. Data pooled from 3 independent experiments and presented as mean $\pm$ SD for all bar graphs. *P<0.05, **P<0.01, anakinra vs untreated (LPS-primed only) and anakinra only *vs* MG132+anakinra. The results show that LPS priming increased the half-life of NLRP3, anakinra reduced it, an activity abrogated in the presence of MG132 but not of leupeptin.

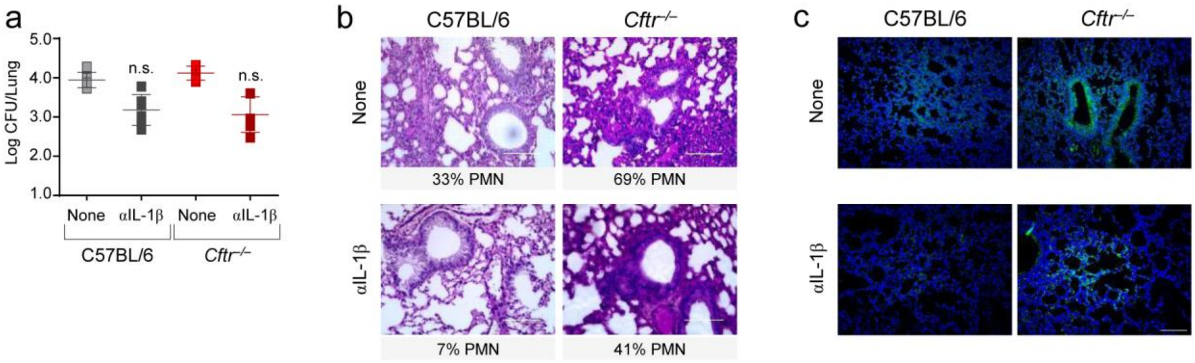

Supplementary Figure 9. C57BL/6 and *Cftr*$^{-/-}$ mice (*n*=6 for all groups) were infected intranasally with live *P. aeruginosa*, treated with anti-IL-1β antibody and assessed at 7dpi for (**a**) bacterial growth (log CFU, mean ±SD) in the lungs (**b**) lung histology (periodic acid-Schiff staining) and (**c**) increased deposition of DNA on lung parenchyma cells on TUNEL staining. Cell nuclei were stained blue with DAPI. Representative images of two independent experiments were acquired using a high-resolution Microscopy Olympus DP71 using a ×20 objective. Scale bar 200 µm. Data pooled from two experiments and presented as mean $\pm$ SD for all bar graphs. n.s., not significant, treated vs untreated mice, Two-way ANOVA Bonferroni post test.

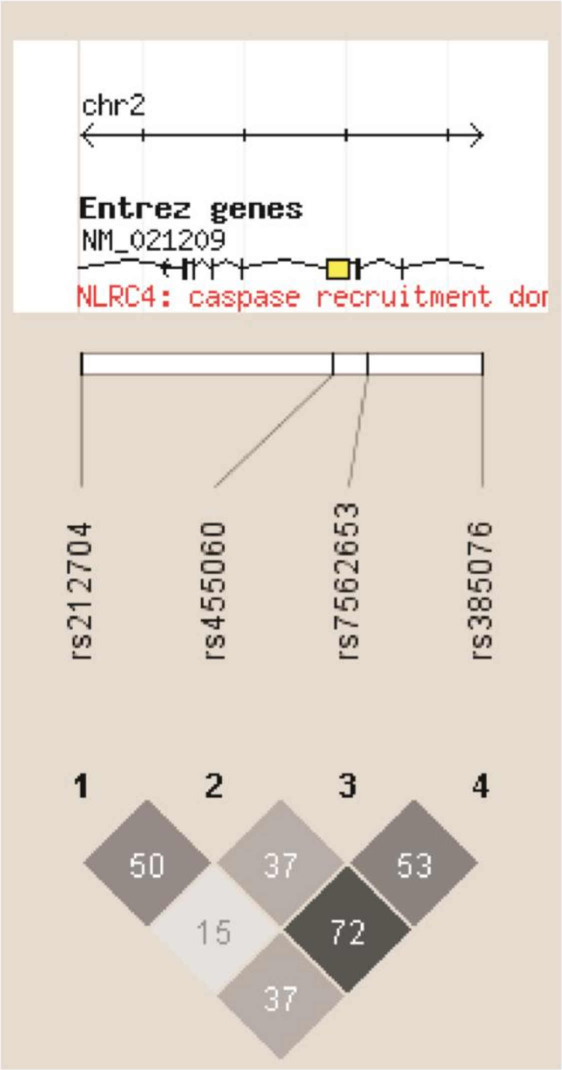

Supplementary Figure 10. Linkage Disequilibrium (LD) plot for *NLRC4* SNPs. LD is expressed in r^2. The plot was generated using Haploview v. 4.2[1]

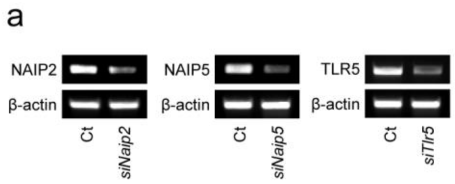

Supplementary Figure 11. Knockdown efficiency. RAW264.7 cells were transfected with siRNA targeting *Naip2*, *Naip5* and TLR5 for 48 h at 37°C in 5% CO_2. The efficiency of gene silencing was assessed by RT-PCR.

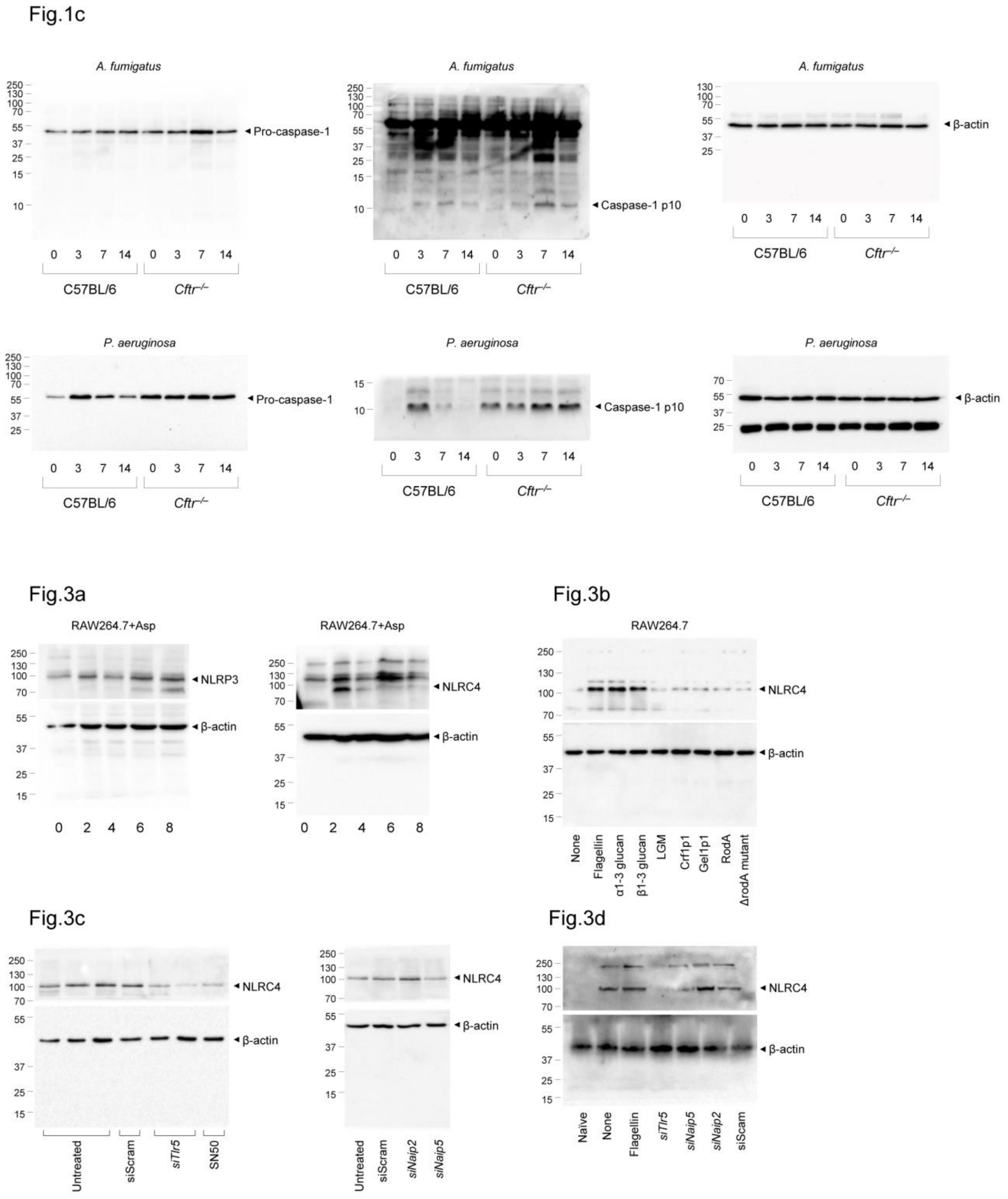

Supplementary Figure 12. Full scan of key blot experiments

Fig.3f

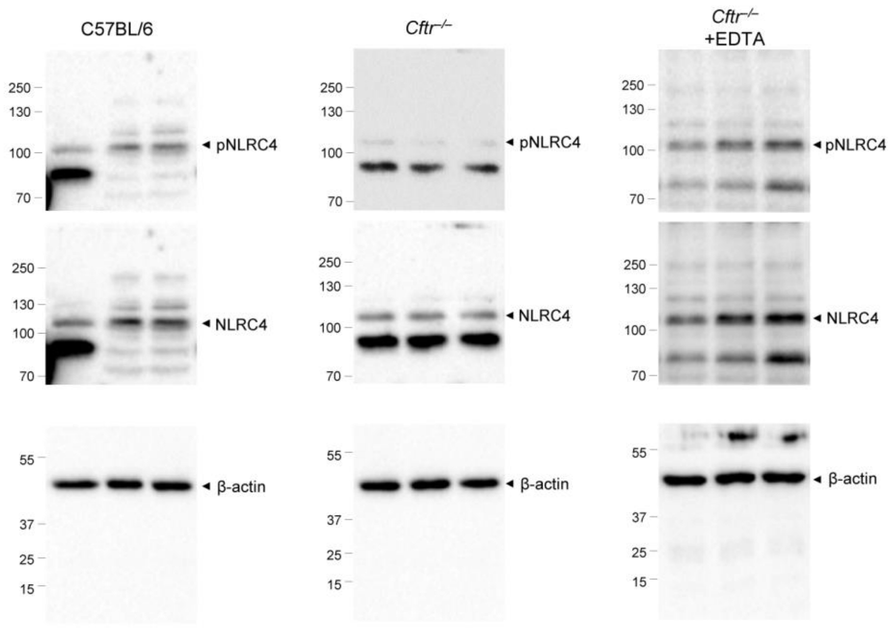

Fig.6d

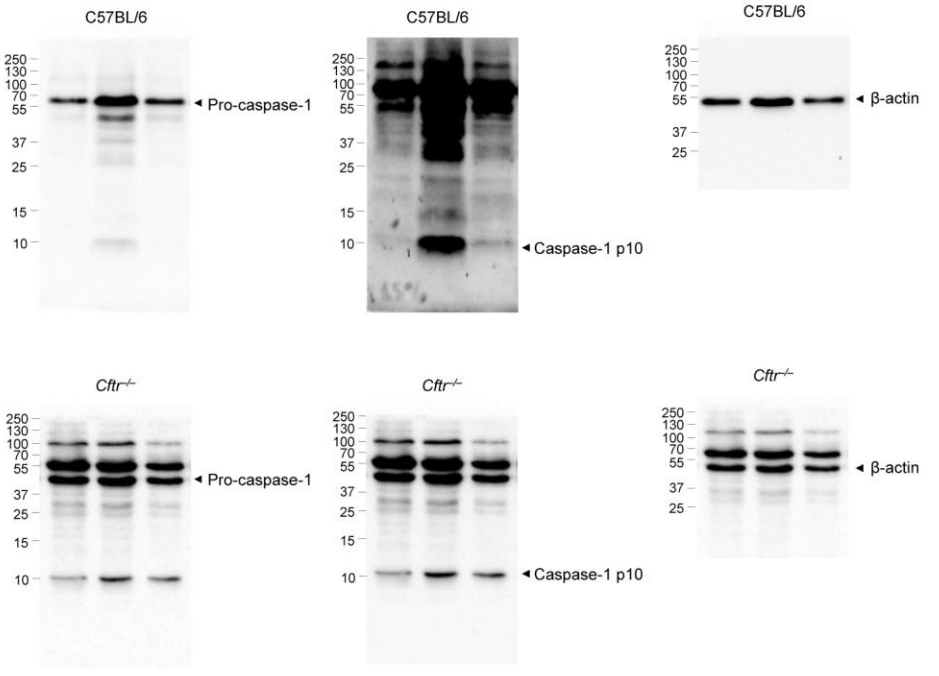

Supplementary Figure 13. Full scan of key blot experiments

Fig.6k

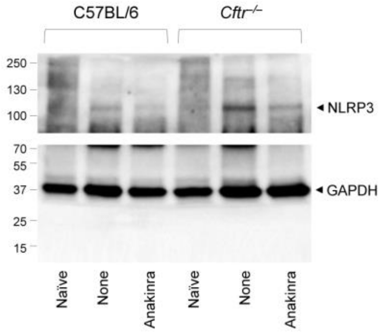

Fig.7b

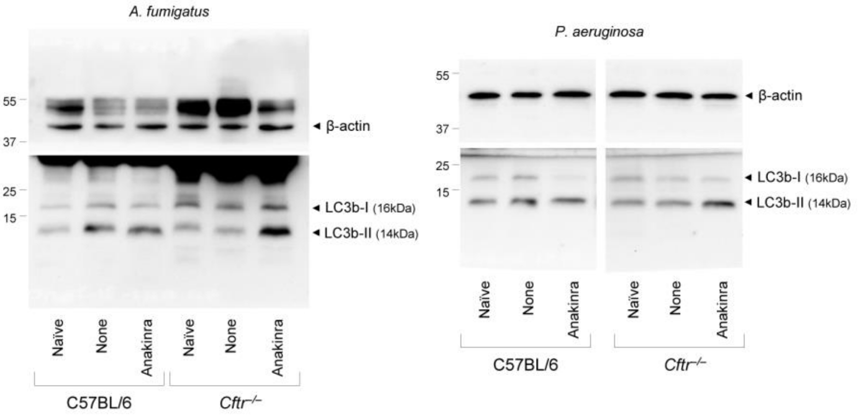

Fig.S5c

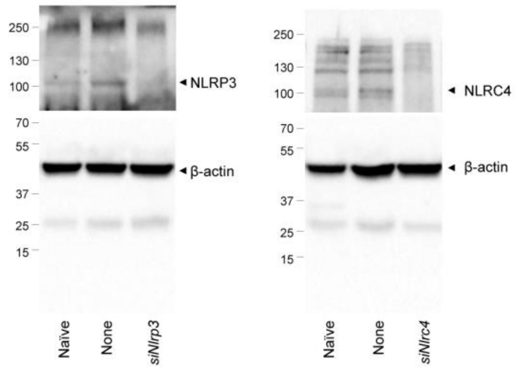

Fig.S8b

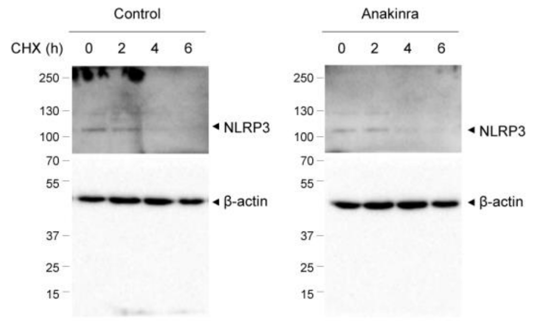

Fig.S8c

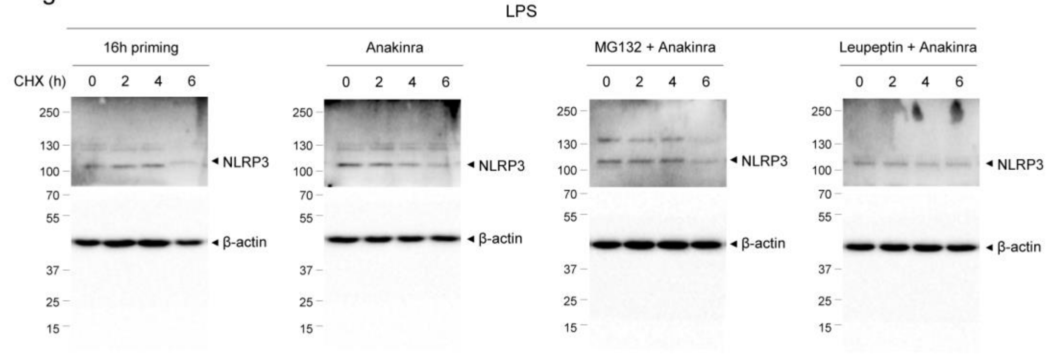

Supplementary Figure 14. Full scan of key blot experiments

Supplementary Table 1. Demographic and clinical characteristics of the CF population study.

		N	Mean ± S.D.	Range
Age in years:		N = 222	14.3 ± 10.9	0.1 - 48
Age at diagnosis in months:		N = 206	31.8 ± 67.0	0 - 408
FEV1, % predicted:		N = 158	84.8 ± 26.7	21.6 – 134.1
FVC, % predicted:		N = 158	90.6 ± 22.3	32.3 – 131
Height, cm:		N = 161	150.3 ± 23.2	95 - 186
Weight, kg:		N = 161	48.2 ± 18.4	13 - 103
BMI:		N = 160	20.3 ± 3.9	13 – 41.8
		**N**	**Percent**	
Sex:	*Male*	142	50.2%	
	Female	141	49.8%	
	M/F ratio	1:1		
CFTR mutation:	*ΔF508 homozygous*	64	22.6%	
	ΔF508 heterozygous	120	42.4%	
	Other	99	35.0%	
Microbial status:	*Pseudomonas* positive	133	47.0%	
	Aspergillus positive	52	18.4%	

FEV1 - forced expiratory volume in the first second; FVC - forced vital capacity; BMI - body mass index; *CFTR* - cystic fibrosis transmembrane conductance regulator. Continuous variables are expressed as mean ± SD.

Supplementary Table 2. Genetic markers genotyped in the CF cohort. Minor Allele Frequency (MAF) and Hardy-Weinberg Equilibrium (HWE) p-value were calculated using Haploview v. 4.2[1]. MAF obtained from the CF cohort was compared to the one reported for Tuscans (TSI) by the 1000 Genomes project[2]. NR=Not Reported.

Gene	Gene map locus	SNP rs ID	Position (bp)	Alleles	HWE p-value	%Genotyped	MAF	MAF TSI
NLRC4	2p22.3	rs212704	chr2:32225279	G:A	1.000	95.7	0.491	0.418
		rs455060	chr2:32250040	T:C	0.228	94.3	0.411	0.520
		rs7562653	chr2:32253560	C:T	0.360	98.9	0.335	0.357
		rs385076	chr2:32264782	C:T	0.219	97.5	0.414	0.449
NLRP3	1q44	rs35829419	chr1:247425556	C:A	0.994	100.0	0.049	0.046
		rs10925026	chr1:247440956	A:C	0.468	93.7	0.395	0.352
IL1B	2q13	rs1143627	chr2:112836810	T:C	0.764	97.9	0.322	0.372
IL1RN	2q13	86bp-int2 VNTR	NR	1:(2+3+4+5)	0.964	100.0	0.349	NR

Supplementary Table 3. Allele association study between *NLRC4* and *Aspergillus* Infection. Association tests were carried out using UNPHASED[3] adjusting for sex and age at sampling.

***NLRC4* SNPs**	**Allele**	**Asp -**	**Asp +**	**OR**	**P-value**
rs212704	A	161 (47.4%)	41 (56.9%)	Reference	
	G	179 (52.7%)	31 (43.1%)	0.633	0.097
rs455060	C	130 (39.6%)	33 (42.3%)	Reference	
	T	198 (60.4%)	45 (57.7%)	0.927	0.778
rs7562653	C	245 (68.8%)	51 (63.8%)	Reference	
	T	111 (31.2%)	29 (36.3%)	1.066	0.813
rs385076	C	208 (59.8%)	46 (59.0%)	Reference	
	T	140 (40.2%)	32 (41.0%)	0.920	0.755

Supplementary Table 4. Haplotype (a), allele (b) and genotype (b) association study between *NLRP3* and *Aspergillus* Infection. Association tests were carried out using UNPHASED[3] adjusting for sex and age at sampling.

a)

	NLRP3 SNPs					
NLRP3* Haplotype	rs35829419	rs10925026	**Asp -**	**Asp +**	**OR**	**P-value**
H1	C	A	192 (57.0%)	44 (57.9%)	Reference	
H2	C	C	128 (38.2%)	27 (35.5%)	0.914	0.691
H3	A	A	14 (4.3%)	5 (6.6%))	1.261	0.637

* Global haplotype test, p-value = 0.854

b)

***NLRP3* SNPs**	**Allele**	**Asp -**	**Asp +**	**OR**	**P-value**
rs35829419	A	18 (5.0%)	6 (7.5%)	Reference	
	C	346 (95.0%)	74 (92.5%)	0.646	0.409
rs10925026	A	206 (61.3%)	49 (64.5%)	Reference	
	C	130 (38.7%)	27 (35.5%)	0.896	0.695

c)

	NLRP3 rs35829419			**A/C**	**C/C**	**Global p-**
**Status**	**A/A (%)**	**A/C (%)**	**C/C (%)**	**OR (p-value)**	**OR (p-value)**	**value**
**Asp -**	---	18 (9.9%)	164 (90.1%)	---	0.626 (0.380)	0.394
**Asp +**	---	6 (15.0%)	34 (85.0%)			
	NLRP3 rs10925026			**A/C**	**C/C**	**Global p-**
**Status**	**A/A (%)**	**A/C (%)**	**C/C (%)**	**OR (p-value)**	**OR (p-value)**	**value**
**Asp -**	60 (35.7%)	86 (51.2%)	22 (13.1%)	0.821 (0.679)	0.854 (0.926)	0.887
**Asp +**	16 (42.1%)	17 (44.7%)	5 (13.2%)			

Supplementary Table 5. Allele and Genotype association study between *IL1B* rs1143627 SNP and *Aspergillus* Infection. Association tests were carried out using UNPHASED[3] adjusting for sex and age at sampling.

Allele	Asp -	Asp +	OR	P-value	
C	119 (33.4%)	25 (32.1%)	Reference		
T	237 (66.6%)	53 (67.9%)	1.051	0.859	
**Genotype**	**Asp -**	**Asp +**	**OR**	**P-value**	**Global p-value**
C/C	21 (11.8%)	3 (7.7%)	Reference		
C/T	77 (43.3%)	19 (48.7%)	1.563	0.633	0.798
T/T	80 (44.9%)	17 (43.6%)	1.385	0.898	

Supplementary Table 6. Allele and Genotype association study between *Il1RN* 86bpIntron2 VNTR and *Aspergillus* Infection. Association tests were carried out using UNPHASED[3] adjusting for sex and age at sampling.

**Allele**	**Asp -**	**Asp +**	**OR**	**P-value**	**Global p-value**
1	234 (64.3%)	52 (65.0%)	Reference		0.924
2	120 (33.0%)	27 (33.8%)	1.025	0.867	
3	1 (0.3%)	---	9.407e-007	0.771	
4	8 (2.2%)	1 (1.3%)	0.547	0.575	
5	1 (0.3%)	---	1.395e-008	0.641	
**Genotype**	**Asp -**	**Asp +**	**OR**	**P-value**	**Global p-value**
1/1	76 (41.8%)	15 (37.5%)	Reference		0.831
1/2	75 (41.2%)	21 (52.5%)	1.411	0.228	
1/4	6 (3.3%)	1 (2.5%)	0.848	0.803	
1/5	1 (0.5%)	---	3.696e-007	0.639	
2/2	21 (11.5%)	3 (7.5%)	0.742	0.497	
2/3	1 (0.5%)	---	5.743e-006	0.771	
2/4	2 (1.1%)	---	2.167e-008	0.473	

Supplementary Table 7. Best models assessed by the GMDR for one to five-way combinations, to test gene-gene interactions in determining *Aspergillus* infection. TBA=Testing Balanced Accuracy, CVC= Cross-Validation Consistency.

Model	TBA	Sign Test (P)	CVC	Odds Ratios (95% C.I.)
NLRC4 rs212704	0.487	4 (0.828)	8/10	1.945 (0.657-5.757)
NLRC4 rs455060 x NLRC4 rs7562653	0.371	2 (0.989)	3/10	2.970 (0.983-8.968)
NLRC4 rs455060 x IL1RN VNTR x IL1B rs1143627	0.521	5 (0.623)	7/10	5.907 (1.908-18.290)
NLRC4 rs212704 x NLRP3 rs10925026 x IL1RN VNTR x IL1B rs1143627	0.486	5 (0.623)	7/10	12.013 (3.669-39.329)
NLRC4 rs455060 x NLRC4 rs7562653 x NLRP3 rs10925026 x IL1RN VNTR x IL1B rs1143627	0.489	5 (0.623)	6/10	41.846 (8.496-206.122)

Supplementary Table 8. Haplotype (A), allele (B) and genotype (C) association study between *NLRC4* and *Pseudomonas* Infection. Association tests were carried out using UNPHASED[3] adjusting for sex and age at sampling.

a)

	NLRC4 SNPs							
NLRC4* Haplotype	rs212704	rs455060	rs7562653	rs385076	**Pseudo -**	**Pseudo +**	**OR**	**P-value**
H1	A	T	C	C	81 (43.1%)	65 (39.9%)	Reference	
H2	G	C	T	T	42 (22.5%)	44 (26.5%)	1.147	0.630
H3	G	T	C	C	18 (9.7%)	27 (16.5%)	1.887	0.065
H4	G	C	C	T	24 (12.6%)	9 (5.6%)	0.629	0.134
H5	G	C	C	C	7 (3.7%)	4 (2.7%)	0.950	0.882
H6	A	T	T	C	3 (1.9%)	3 (2.1%)	1.452	0.601
H7	A	C	T	T	6 (3.2%)	5(3.1%)	1.004	0.353
H8	A	T	T	T	2 (1.1%)	1 (0.6%)	0.351	1.000
H9	A	T	C	T	2 (1.1%)	---	0.000	0.114
H10	G	C	T	C	---	1 (1.1%)	3.789e+008	0.074
H11	A	C	C	C	2 (1.1%)	---	8.543e-007	0.372
H12	G	T	T	T	---	2 (1.3%)	4.78e+009	1.000

* Global haplotype test, p-value = 0.148

b)

NLRC4 SNPs	Allele	Pseudo -	Pseudo +	OR	P-value
rs212704	A	107 (51.9%)	84 (44.7%)	Reference	
	G	99 (48.1%)	104 (55.3%)	1.299	0.219
rs455060	C	89 (42.4%)	69 (38.8%)	Reference	
	T	121 (57.6%)	109 (61.2%)	1.187	0.432
rs7562653	C	160 (72.1%)	123 (62.8%)	Reference	
	T	62 (27.9%)	73 (37.2%)	1.703	0.193
rs385076	C	128 (59.8%)	114 (58.8%)	Reference	
	T	86 (40.2%)	80 (41.2%)	0.939	0.768

c)

	NLRC4 rs212704			A/G	G/G	Global p-
**Status**	**A/A (%)**	**A/G (%)**	**G/G (%)**	**OR (p-value)**	**OR (p-value)**	**value**
**Pse -**	25 (24.3%)	57 (55.3%)	21 (20.4%)	0.800 (0.105)	1.656 (0.056)	0.136
**Pse +**	20 (21.3%)	44 (46.8%)	30 (31.9%)			
	***NLRC4* rs455060**			**C/T**	**T/T**	**Global p-**
**Status**	**C/C (%)**	**C/T (%)**	**T/T (%)**	**OR (p-value)**	**OR (p-value)**	**value**
**Pse -**	16 (15.2%)	57 (54.3%)	32 (30.5%)	1.206 (0.763)	1.48 (0.455)	0.699
**Pse +**	10 (11.2%)	49 (55.1%)	30 (33.7%)			
	***NLRC4* rs7562653**			**C/T**	**T/T**	**Global p-**

Status	C/C (%)	C/T (%)	T/T (%)	OR (p-value)	OR (p-value)	value
**Pse -**<br>**Pse +**	57 (51.4%)<br>37 (37.8%)	46 (41.4%)<br>49 (50.0%)	8 (7.2%)<br>12 (12.2%)	1.295 (0.648)	1.950 (0.283)	0.400
**Status**	***NLRC4* rs385076**<br>**C/C (%)**	**C/T (%)**	**T/T (%)**	**C/T**<br>**OR (p-value)**	**T/T**<br>**OR (p-value)**	**Global p-value**
**Pse -**<br>**Pse +**	36 (33.6%)<br>33 (34.0%)	56 (52.3%)<br>48 (49.5%)	15 (14.0%)<br>16 (16.5%)	0.768 (0.396)	0.966 (0.274)	0.694

Supplementary Table 9. Haplotype (A), allele (B) and genotype (C) association study between *NLRP3* and *Pseudomonas* Infection. Association tests were carried out using UNPHASED[3] adjusting for sex and age at sampling.

a)

	NLRP3 SNPs					
NLRP3* Haplotype	rs35829419	rs10925026	**Pseudo -**	**Pseudo +**	**OR**	**P-value**
H1	C	A	122 (57.0%)	104 (57.7%)	Reference	
H2	C	C	78 (36.5%)	69 (38.4%)	1.091	0.396
H3	A	A	14 (2.8%)	5 (2.8%)	0.347	0.127
H4	A	C	---	2 (1.1%)	4.514e+201	0.583

* Global haplotype test, p-value = 0.156

b)

***NLRP3* SNPs**	**Allele**	**Pseudo -**	**Pseudo +**	**OR**	**P-value**
rs35829419	A	15 (6.8%)	9 (4.4%)	Reference	
	C	207 (93.2%)	195 (95.6%)	1.667	0.258
rs10925026	A	136 (63.6%)	109 (60.6%)	Reference	
	C	78 (36.5%)	71 (39.4%)	1.221	0.362

c)

	***NLRP3* rs35829419**			**A/C**	**C/C**	**Global p-value**
**Status**	**A/A (%)**	**A/C (%)**	**C/C (%)**	**OR (p-value)**	**OR (p-value)**	
**Pse -**	---	15 (13.5%)	96 (86.5%)	---	1.721 (0.243)	0.240
**Pse +**	---	9 (8.8%)	93 (91.2%)			
	***NLRP3* rs10925026**			**A/C**	**C/C**	**Global p-value**
**Status**	**A/A (%)**	**A/C (%)**	**C/C (%)**	**OR (p-value)**	**OR (p-value)**	
**Pse -**	42 (39.3%)	52 (48.6%)	13 (12.1%)	1.199 (0.827)	1.577 (0.443)	0.641
**Pse +**	32 (35.6%)	45 (50.0%)	13 (14.4%)			

Supplementary Table 10. Allele and Genotype association study between *IL1B* rs1143627 SNP and *Pseudomonas* Infection. Association tests were carried out using UNPHASED[3] adjusting for sex and age at sampling.

Allele	Pseudo -	Pseudo +	OR	P-value	
C	75 (34.1%)	63 (32.1%)	Reference		
T	145 (65.9%)	133 (67.9%)	1.060	0.790	
**Genotype**	**Pseudo -**	**Pseudo +**	**OR**	**P-value**	**Global p-value**
C/C	14 (12.7%)	9 (9.2%)	Reference		
C/T	47 (42.7%)	45 (45.9%)	1.348	0.722	0.836
T/T	49 (44.6%)	44 (44.9%)	1.271	0.999	

Supplementary Table 11. Allele and Genotype association study between *Il1RN* 86bpIntron2 VNTR and *Pseudomonas* Infection. Association tests were carried out using UNPHASED[3] adjusting for sex and age at sampling.

Allele	Pseudo -	Pseudo +	OR	P-value	Global p-value
1	141 (63.5%)	133 (65.2%)	Reference		0.677
2	75 (33.8%)	67 (32.8%)	0.968	0.936	
3	1 (0.5%)	---	1.112e-008	0.384	
4	5 (2.3%)	3 (1.5%)	0.622	0.548	
5	---	1 (0.5%)	4.402e+010	0.468	
**Genotype**	**Pseudo -**	**Pseudo +**	**OR**	**P-value**	**Global p-value**
1/1	45 (40.5%)	43 (42.2%)	Reference		0.827
1/2	47 (42.3%)	44 (43.1%)	0.996	0.860	
1/4	4 (3.6%)	2 (2.0%)	0.453	0.386	
1/5	---	1 (1.0%)	2.357e+009	0.466	
2/2	13 (11.7%)	11 (10.8%)	0.898	0.857	
2/3	1 (1.0%)	---	3.514e-008	0.382	
2/4	1 (1.0%)	1 (1.0%)	1.577	0.745	

Supplementary References

1. Barrett, J.C., Fry, B., Maller, J. & Daly, M.J. Haploview: analysis and visualization of LD and haplotype maps. *Bioinformatics* **21**, 263-265 (2005).
2. Genomes Project, C.*, et al.* An integrated map of genetic variation from 1,092 human genomes. *Nature* **491**, 56-65 (2012).
3. Dudbridge, F. Pedigree disequilibrium tests for multilocus haplotypes. *Genet Epidemiol* **25**, 115-121 (2003).
